# A chromosome‐scale assembly of allotetraploid *Brassica juncea* (AABB) elucidates comparative architecture of the A and B genomes

**DOI:** 10.1111/pbi.13492

**Published:** 2020-12-30

**Authors:** Kumar Paritosh, Satish Kumar Yadava, Priyansha Singh, Latika Bhayana, Arundhati Mukhopadhyay, Vibha Gupta, Naveen Chandra Bisht, Jianwei Zhang, David A. Kudrna, Dario Copetti, Rod A. Wing, Vijaya Bhasker Reddy Lachagari, Akshay Kumar Pradhan, Deepak Pental

**Affiliations:** ^1^ Centre for Genetic Manipulation of Crop Plants University of Delhi South Campus New Delhi India; ^2^ Department of Genetics University of Delhi South Campus New Delhi India; ^3^ National Institute of Plant Genome Research New Delhi India; ^4^ Arizona Genomics Institute School of Plant Sciences The University of Arizona Tucson AZ USA; ^5^ AgriGenome Labs Pvt Ltd, Genome Valley Shamirpet Hyderabad India

**Keywords:** *Brassica juncea*, oilseed mustard, *Brassica nigra*, long‐read sequencing, genome assembly, gene blocks, evolution, breeding

## Abstract

*Brassica juncea* (AABB), commonly referred to as mustard, is a natural allopolyploid of two diploid species—*B. rapa* (AA) and *B. nigra* (BB). We report a highly contiguous genome assembly of an oleiferous type of *B. juncea* variety Varuna, an archetypical Indian gene pool line of mustard, with ~100× PacBio single‐molecule real‐time (SMRT) long reads providing contigs with an N50 value of >5 Mb. Contigs were corrected for the misassemblies and scaffolded with BioNano optical mapping. We also assembled a draft genome of *B. nigra* (BB) variety Sangam using Illumina short‐read sequencing and Oxford Nanopore long reads and used it to validate the assembly of the B genome of *B. juncea*. Two different linkage maps of *B. juncea*, containing a large number of genotyping‐by‐sequencing markers, were developed and used to anchor scaffolds/contigs to the 18 linkage groups of the species. The resulting chromosome‐scale assembly of *B. juncea* Varuna is a significant improvement over the previous draft assembly of *B. juncea* Tumida, a vegetable type of mustard. The assembled genome was characterized for transposons, centromeric repeats, gene content and gene block associations. In comparison to the A genome, the B genome contains a significantly higher content of LTR/Gypsy retrotransposons, distinct centromeric repeats and a large number of *B. nigra* specific gene clusters that break the gene collinearity between the A and the B genomes. The *B. juncea* Varuna assembly will be of major value to the breeding work on oleiferous types of mustard that are grown extensively in south Asia and elsewhere.

## Introduction

Genus Brassica contains some of the most important oilseed and vegetable crops that are grown worldwide. Nagaharu (1935), based on cytogenetic studies, proposed a model on the relationship of six key species of the genus. The model, known as ‘U’s triangle, has three diploid species—*B. rapa* (Bra, AA, *n* = 10), *B. nigra* (Bni, BB, *n* = 8) and *B. oleracea* (Bol, CC, *n* = 9) on the nodes and three allotetraploids—*B. juncea* (Bju, AABB, *n* = 18), *B. napus* (Bna, AACC, *n* = 19) and *B. carinata* (Bca, BBCC, *n* = 17) at the coordinates. *B. rapa* was the first of the six species to be sequenced (Wang *et al*., 2011) followed by *B. oleracea* (Liu *et al*., 2014), *B. napus* (Chalhoub *et al*., 2014) and *B. juncea* (Yang *et al*., 2016). Draft genomes of *B. rapa* (estimated genome size ~485 Mb), *B. oleracea* (~630 Mb) and *B. napus* (~1130 Mb) were assembled with short‐read, high‐throughput Illumina technology with some limited Sanger sequencing (Chalhoub *et al*., 2014; Liu *et al*., 2014; Wang *et al*., 2011). A draft sequence of a vegetable type of *B. juncea* variety Tumida (genome size ~922 Mb) was assembled using ~176× Illumina reads, gap filling with 12× PacBio single‐molecule real‐time (SMRT) sequences and long‐range scaffolding with BioNano optical mapping (Yang *et al*., 2016).

The long‐read third‐generation technologies such as SMRT sequencing by PacBio and Nanopore sequencing combined with long‐range scaffolding technologies have significantly improved genome assemblies providing higher levels of contiguity and more extensive coverage of repeat sequences, transposable elements, and centromeric and telomeric regions (Li *et al*., 2018; Van‐Dijk *et al*., 2018). Amongst the Brassica species, assembly of *B. rapa* Chiifu has been improved by additional data from ~57× SMRT sequencing, BioNano optical mapping and Hi‐C reads (Zhang *et al*., 2018). Highly contiguous de novo assemblies have been generated for two new lines of *B. rapa* and *B. oleracea* by Nanopore long‐read sequencing and optical mapping (Belser *et al*., 2018).

We report here reference genome assembly of an oleiferous type of *B. juncea*—variety Varuna, an archetypical line of the Indian gene pool of mustard, with ~100× SMRT coverage using PacBio Sequel chemistry and long‐range scaffolding with BioNano optical mapping. To facilitate and validate the assembly of the two constituent genomes—A and B of *B. juncea,* a draft genome of an Indian gene pool line of *B. nigra* (variety Sangam) was assembled using Illumina shotgun sequencing and gap filling with Oxford Nanopore (ONT) long‐read sequences. Sequence assemblies were further validated and assigned to pseudochromosomes with three new genetic maps containing a large number of GBS (genotyping by sequencing) markers.

Oleiferous *B. juncea* types are extensively grown in the dryland area of south Asia and are a potential crop for the other low moisture availability regions of the world. In India alone, *B. juncea* is grown as an oilseed crop in 5–6 million hectares of land during the winter season (Jat *et al*., 2019). We chose variety Varuna for genome sequencing as it is one of the most extensively grown mega‐variety of mustard released by the public‐funded breeding programmes in India (Chauhan *et al*., 2011). The genome assembly reported here will be of major value to the breeding programmes and would allow better utilization of extensive variability available within the different gene pools of *B. juncea*.

## Results

### Genome assembly

To assemble the genome of *B. juncea* variety Varuna (AABB, genome size ~922 Mb reported by Yang *et al*., 2016), three different experimental activities were initiated concurrently. In the first activity, high molecular weight DNA isolated from *B. juncea* variety Varuna was subjected to SMRT sequencing on the PacBio RSII platform. A total of 9 735 857 reads, with an N50 value of ~15.5 kb, were obtained—providing ~100× coverage of the genome (Table S1). The reads were assembled into 1253 contigs with an N50 value of ~5.7 Mb using Canu assembler (Table 1). *B. juncea* genome was also sequenced on an Illumina HiSeq platform to obtain ~40× coverage of the genome (Table S2). Around 98.5% of the short reads could be mapped to the SMRT sequencing‐based contigs, indicating that the long‐read sequences had covered most of the genomic regions.

**Table 1:** Genome assembly statistics of *Brassica juncea* variety Varuna

PacBio	✓	✓	✓	✓	✓
BioNano		✓	✓	✓	✓
Linkage Map			✓	✓	✓
	*B. juncea* (AABB, 2*n* = 18)			*B. juncea* A	*B. juncea* B
Total assembly size	869 539 390	–	–	–	–
Number of contigs	1253	–	–	–	–
Longest contig	35 843 917	–	–	–	–
N50 contig length	5 734 093	–	–	–	–
Number of scaffolds	–	61	–	–	–
Longest scaffold	–	72 092 522	–	–	–
N50 scaffold length	–	33 612 814	–	–	–
Unscaffolded contigs	–	714	–	–	–
Number of pseudochromosomes/LGs	–	–	18	10	8
Scaffolds assigned to LGs	–	–	61	42	19
Contigs assigned to LGs	–	–	9	9	0
Unassigned contigs to LGs	–	–	705	–	–
Unassigned scaffolds to LGs	–	–	4	–	–
Length of assigned sequences to LGs	–	–	840 173 505	333 021 048	507 152 457
Length of unassigned sequences to LGs	–	–	35 468 516	–	–
Missing bases	0	–	3.43	5.72	1.67

In the second activity, a doubled haploid (DH) line of *B. nigra* (BB) variety Sangam, genome size ~591 Mb (Yang *et al*., 2016), was sequenced using the Illumina HiSeq platform. Around 110× coverage was obtained by sequencing paired‐end (PE) libraries of three different insert sizes (Table S3a). An additional 2.8 Gb sequence data were obtained from three different mate‐pair (MP) libraries (Table S3b). The PE reads were assembled to obtain 57 941 contigs with an N50 value of ~34.6 kb. These contigs were scaffolded using ~14× Oxford Nanopore long‐read sequence data (Table S3c) followed by MP library data. These processes resulted in an assembly consisting of 18 996 scaffolds with an N50 value of ~85.9 kb covering around 470.8 Mb of the *B. nigra* genome (Table S4). Around 346.9 Mb of *B. nigra* genome could be assembled into eight pseudochromosomes (Table S4).

In the third activity, three genetic maps were developed—two for *B. juncea* and one for *B. nigra*. An earlier developed genetic map of *B. juncea* using an F_1_DH population derived from a cross of Varuna and Heera (an east European gene pool line)—named the VH population (Pradhan *et al*., 2003), which contained 833 intron polymorphic (IP), genic SSR and SNP markers (Panjabi *et al*., 2008; Paritosh *et al*., 2014), was further enriched by the addition of 2947 GBS markers using *Sph*I‐*Mluc*I enzyme combination (Tables S5 and S6; Figure S1). A second F_1_DH population (named TuV) was developed in this study from a cross between Tumida and Varuna. A population of 119 individuals was first mapped with 524 IP and SSR markers that were common with the VH population—subsequently, 8517 GBS markers, using the *Hinf*I‐*Hpy*CH4IV enzyme combination, were added for a more extensive coverage (Tables S7 and S8; Figure S2). A third F_1_DH mapping population was developed from a cross between *B. nigra* variety Sangam (an Indian gene pool line) and line 2782 (an east European type line). This map contained, besides a common set of IP and genic SSR markers with the VH population, an additional 2723 GBS markers (Tables S9 and S10; Figure S3). The genetic map of *B. nigra* was used for assigning scaffolds to the eight LGs (Table S4; Figure S4).

A comparison was made between the 1253 SMRT based contigs of *B. juncea*, *B. rapa* (A) genome sequences (Wang *et al*., 2011), and the 18 996 assembled scaffolds of *B. nigra* (B) to identify the A and B subgenome specific contigs in the Varuna assembly. Predicted protein encoding genes in the assembled contigs of *B. juncea*, and scaffolds of *B. nigra* were subjected to a reciprocal protein blast to identify the best matching homologous regions. *B. juncea* contigs with ≥95% genes showing the best matching orthologous genes to either of the two parental genomes were grouped as the A or B genome‐specific contigs. Of the 1253 contigs, 691 were found to belong to the B genome and 505 contigs were found to be specific to the A genome; 57 contigs could not be specified to either of the two genomes. Around eighteen misassemblies were noticed—these had mostly arisen due to the coalescence of highly conserved syntenic regions. These misassemblies were confirmed by the high‐density genetic map of the VH population. However, no corrections were made based on this analysis.

Independent of the above‐described analysis, the 1253 SMRT contigs of *B. juncea* were subjected to hierarchical mapping using three different optical maps. Two maps were developed with NLRS (Nicks, Labels, Repairs and Stains) based labelling using *BssS*I and *BspQ*I enzymes and one with DLS (Direct Label and Stain) labelling using the DLE‐1 enzyme (Table S11a; Figure S5). Corrections were made at each mapping stage, and corrected sequences were mapped again on the consensus maps. A total of 45 contigs were identified with misassemblies. These included the eighteen misassemblies that were identified earlier. All the misassemblies were curated by breaking the misassembled regions and aligning the edited contigs again to the consensus maps. The final assembly after corrections and multiple rounds of scaffolding consisted of 61 scaffolds and 714 unscaffolded contigs/fragments (Table 1). The total size of the assembled scaffolds was ~847.8 Mb with an N50 value of ~33.6 Mb. The unscaffolded contigs/fragments constituted ~36.4 Mb of the genome with an N50 value of ~55.6 kb. Scaffolds were polished using Illumina short reads in three iterated rounds of the Pilon program (Walker *et al*., 2014). A total of 63 304 indels and 16 906 SNVs were corrected in the process (Table S11b).

**Figure 1:**
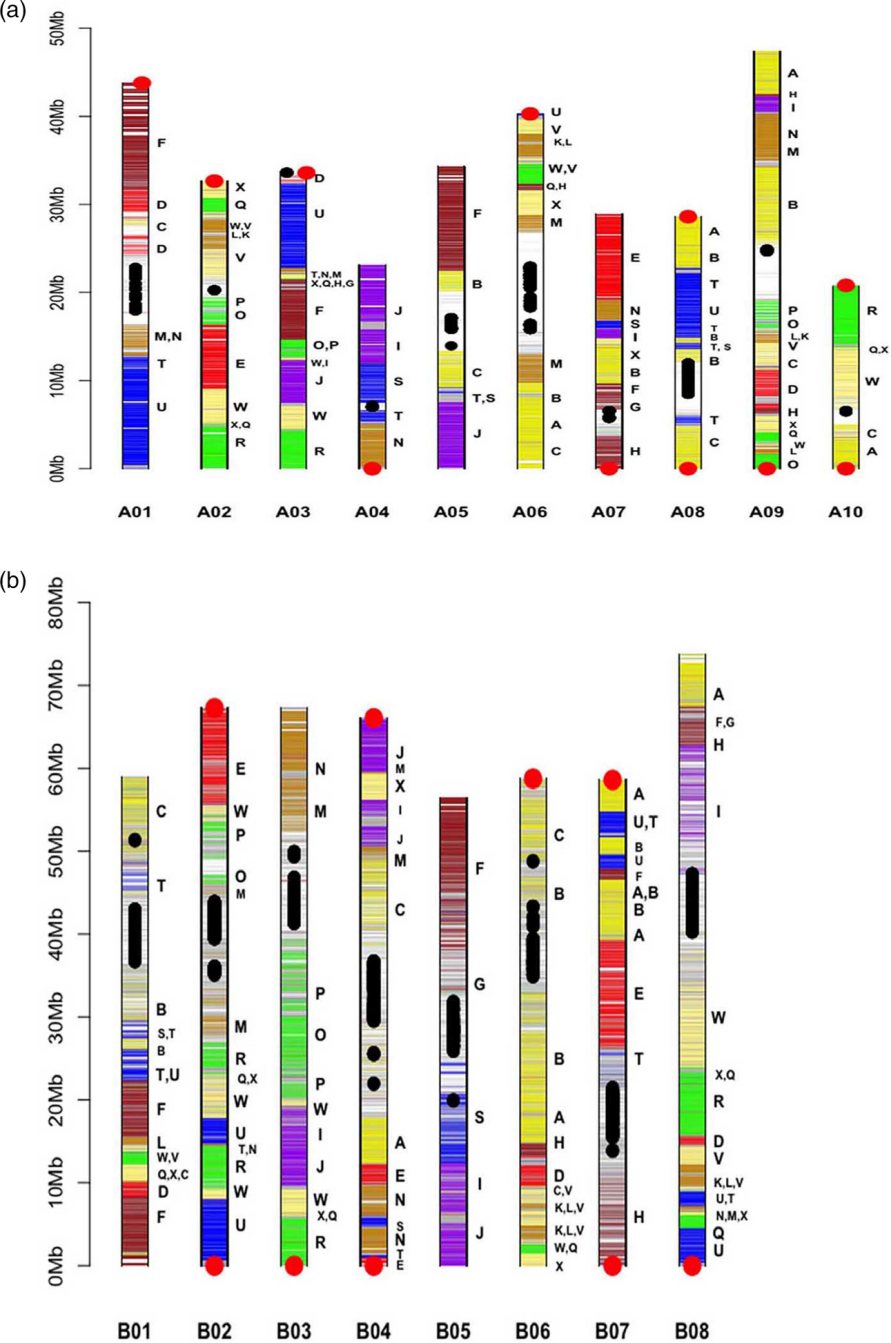
Graphic representation of the *Brassica juncea* A genome (a) and B genome (b) pseudochromosomes. Horizontal bars represent the predicted genes. Different regions of the pseudochromosomes have been assigned to gene blocks based on synteny with *Arabidopsis thaliana* gene blocks (A–X), as defined by Schranz *et al*. (2006). The colour code used for *B. juncea* gene blocks is as has been used for the gene blocks in *A. thaliana*. Centromeric repeats are represented as black dots and telomeric repeats as red dots.

The VH genetic map was used to assign the scaffolds and contigs to 18 linkage groups (LGs) of *B. juncea*. Fifty‐six of the 61 scaffolds and nine of the 714 contigs could be assigned to the 18 pseudochromosomes—which were designated as BjuA01–BjuA10 and BjuB01–BjuB08 (Panjabi *et al*., 2008; Paritosh *et al*., 2014; Figure 1; Table S12). Three of the pseudochromosomes were covered with only one scaffold, four with two scaffolds, six with three and the rest with four or more scaffolds, and a few contigs—the maximum number being ten for BjuA01. GBS markers, 2947 in number, mapped on the VH population were used to compare their position on the genetic map with their physical location on the pseudochromosomes. A very high correlation was observed between the marker order on the genetic map and the physical position of the GBS tags on the pseudochromosomes (Figure S6). To further validate the assembly, we used the TuV population genetic map, which contained 8517 GBS markers assigned to the 18 LGs of *B. juncea*. The order of the dispersed GBS markers on the TuV genetic map was invariably found to be correlated with the order of their sequences on the pseudochromosomes (Figure S7). Based on the genetic marker data, two more scaffolds could be assigned to the pseudochromosomes (Table S12). At the end of all the steps, from the overall genome assembly consisting of 58 scaffolds and 714 contigs/fragments—58 scaffolds and nine contigs could be assigned to the 18 pseudochromosomes; three scaffolds and 705 contigs remained unassigned (Table 1). The size of the assigned sequences was calculated to be around 840.2 Mb (~91.2% of the estimated genome content). About 35.4 Mb of the sequenced genome (~3.7% of the total genome) remained unassigned.

We carried out orthologue tagging of the *B. juncea* Varuna genes with the previously assembled *B. rapa* Chiifu (V1.5) (Wang *et al*., 2011) and *B. juncea* Tumida (V1.1) (Yang *et al*., 2016) assemblies; many misassembled regions in the previous assemblies could be identified (Figure S11a). A total of 35 644 (88.3%) and 1802 (92.5%) of the contigs/scaffolds that remained unassigned in the previous *B. rapa* (V1.5) and *B. juncea* Tumida (V1.1) assemblies, respectively, could be placed on the 18 pseudochromosomes of the *B. juncea* Varuna. Misassemblies were found to be more pronounced in the assembly of the B genome of *B. juncea* Tumida. A comparison of the chromosomes level assembly of *B. juncea* Varuna genome with the assembly earlier reported for *B. juncea* Tumida (V1.1) (Yang *et al*., 2016), and its improved version V1.5 (Yang *et al*., 2018) showed a high level of contiguity and lesser number of gaps in the Varuna assembly (Table S13). As compared to the 13.17% and 21.65% of average gaps present in A and B genomes of *B. juncea* (V1.5), only 5.72% and 1.67% of gaps were observed in the corresponding Varuna genome assembly. The coverage achieved for the B genome in the present study was significantly higher—~507.1 Mb, as compared to the earlier reported coverage of ~395.9 Mb in V1.1 (Yang *et al*., 2016) and ~377.6 Mb in V1.5 (Yang *et al*., 2018) (Table S13).

### Genome annotations

#### *Transposable elements*

*B. juncea* Varuna genome assembly was annotated for three components—transposable elements, centromeres and genes. Transposable elements (TEs) were identified by structure and similarity using a *de novo* prediction approach (Methods for the details). This exercise generated a database of 1590 consensus repeat sequences that were merged with the *Arabidopsis thaliana* (At) TE database to annotate TEs in the assembled pseudochromosomes of the A and B genomes, separately. Repeats were broadly classified under three major categories—retrotransposons, DNA transposons and other repeats, and further subclassified (Table S14). Around 385 Mb (~45.8%) of the assembled genome of Varuna was found to be constituted of TEs. The B genome had a higher repeat content ~259 Mb (~51%) as compared to the A genome—113 Mb (~33.9%). Retrotransposons—LTR/Copia and LTR/Gypsy, were the predominant TEs present in the Varuna genome. Both these types showed significant expansion in the B genome. LTR/Copia constituted ~4.4% (~14 Mb) of the A genome; in comparison, these elements constituted around ~9% (46 Mb) of the B genome. The expansion of LTR/Gypsy was most pronounced in the B genome, ~21.5% (~109 Mb) in comparison to ~7.5% (~25 Mb) in the A genome (Table S14; Figure S8).

### *Centromeric regions*

Candidate centromeric regions were identified from the correlation plots between GBS markers on the genetic maps and the physical positions of their respective tags on the pseudochromosomes (Figures S6 and S7); regions with significantly low recombination frequencies were marked as potential centromeric regions and analysed for repeats that were absent from other parts of the pseudochromosomes (Methods for the details). CentBr1 and CentBr2, identified as centromere‐specific sequences in *B. rapa* (A genome) and *B. oleracea* (C genome) in earlier studies (Liu *et al*., 2014; Wang *et al*., 2011), were found to be present in the A genome of *B. juncea* but absent from the centromeric regions of all the pseudochromosomes of the B genome. Three new A genome‐specific centromeric repeats and seven B genome‐specific repeats were identified (Tables S15 and S16; Figures S9 and S10). Further, the centromeric/pericentromeric regions of all the B genome pseudochromosomes contained a much higher content of TEs as compared to such areas in the A genome (Table S17; Figure S8).

### *Gene annotation*

For gene annotation, we performed RNA‐seq analysis on poly‐A enriched RNA isolated from seedling and young inflorescence tissues of Varuna on a PacBio RSII platform. A total of 40 208 full‐length high‐quality sequences were obtained (details provided in File S1). Genome sequences assigned to the 18 pseudochromosomes of Varuna were repeat‐masked (see Methods) and analysed for gene content with Augustus software, using 543 randomly selected full‐length CDS obtained from the PacBio based RNA‐seq analysis as the training data set. A total of 101 959 genes were predicted of which 46 381 in the A genome and 55 578 in the B genome were assigned to the individual chromosomes (Table S18). The earlier Tumida genome assembly, predominantly based on short‐read sequencing, had reported only 80 050 genes (Yang *et al*., 2016). We validated the genes predicted in the Varuna assembly by comparing these with the non‐redundant proteins in the UniProt plant database. This analysis could validate around 93% of the predicted genes. Predicted genes were also confirmed by the Iso‐seq transcriptome analysis and short‐read RNA‐seq data generated for *B. juncea* in some other studies (Bhardwaj *et al*., 2015; Paritosh *et al*., 2014; Sharma *et al*., 2015) (File S1). A total of 79 108 genes were found to express in one or the other study—37 256 (~80%) and 41 853 (~75.3%) in the A and B genomes, respectively.

We also carried out RNA‐seq of *B. nigra* Sangam using RNA isolated from the seedling and young inflorescence tissues on Illumina and Roche sequencing platforms (File S1). For the *de novo* prediction of the genes in the assembled *B. nigra* genome, the eight pseudochromosomes and unassigned scaffolds of *B. nigra* were used for gene prediction with Augustus software using the same training data set as used for *B. juncea* gene prediction. A total of 46 227 genes were predicted, of which 35 981 were found to express in the RNA‐seq carried out on *B. nigra* tissues (File S1). All the expressed genes in *B. nigra* were found to be present in the annotated B genome of *B. juncea* Varuna.

A data file was generated for *B. juncea* with information on the physical position of every predicted gene on the *B. juncea* 18 pseudochromosomes—BjuA01–BjuA10 and BjuB01–BjuB08 (Table S18). This data set also contains information on the expression status of each of the predicted gene and its closest orthologue in *B. rapa* Chiifu and the model species At based on protein similarity. Genes were marked based on 24 ancestral genomic blocks (A‐X) defined for the At genome (Schranz *et al*., 2006; Figure 1; Table S18).

We compared the assembly of the A genome of *B. juncea* Varuna with the most recent *B. rapa* Chiifu assembly (V3.0) (Zhang *et al*., 2018) that was improved with PacBio long reads and Hi‐C analysis. A gene‐to‐gene correlation analysis based on the least Ks values showed very high collinearity between the BjuA and BraA genomes (Figure S11b). The updated genome of *B. rapa* V3.0 reported a gene content of 45 985 genes, whereas our analysis has predicted 46 381 genes in the BjuA genome. This analysis confirmed that there have been no significant changes in the BjuA genome after the allopolyploidy event and the two A genomes can be taken as equivalent. A similar comparison between the BjuB genome and a new assembly of the BniB genome assembled by using Oxford Nanopore long reads and optical mapping (Paritosh *et al*., 2020) has also shown similar gene content (57 249 in BniB vs 57 084 in BjuB) and arrangement in the two B genomes (Figure S11b). Our analysis shows that there are no significant changes in the two subgenomes of *B. juncea* (BjuA and BjuB) after polyploidization in comparison to their corresponding progenitor diploid genomes (BraA and BniB). No exchanges (≥5 consecutive genes) were observed between the homoeologous regions of the BjuA and BjuB genomes (Figure S11b).

Orthologous gene groups between the A and B genomes of *B. juncea* were identified based on protein similarity using OrthoFinder (Emms and Kelly, 2015). A total of 19 404 orthologous groups were identified between the A and B genomes; 39 383 genes of A had orthologues in B, and 42 727 genes of B had orthologues in the A genome. Further, 8934 A genome‐specific and 19 193 B genome‐specific genes were identified (Blastp analysis with an e‐value cut‐off of 1e‐05, coverage >80%). A total of 2937 A genome‐specific genes and 12 262 of B genome‐specific genes were observed to express in the RNA‐seq data of *B. juncea* and *B. nigra* (Figures S12 and S13). We checked whether the genome‐specific genes were dispersed or present in clusters. A total of 98 gene clusters with ≥10 species‐specific genes were identified in the B genome, whereas only 15 such clusters were observed in the A genome.

### Evolution of the A and B genomes of *B. juncea*

Diploid taxa belonging to the tribe Brassicaceae, including those containing the three genomes—A, B and C of U's triangle, have originated through genome triplication—the ***b*** event (Lysak and Koch, 2011; Lysak *et al*., 2005; Panjabi *et al*., 2008; Parkin *et al*., 2005; Schranz *et al*., 2006). The three constituent paleogenomes of the A genome showed differential gene retention/loss pattern and were described as LF_A_ (least fragmented), MF1_A_ (moderately fragmented) and MF2_A_ (most fragmented) (Wang *et al*., 2011). We studied gene fractionation in the A and B genomes of *B. juncea* vis‐a‐vis the At genome (Table S19). A similar trend for gene fractionation was observed in the A and B genomes of *B. juncea* (Table S19; Figure S14)—hence, the three paleogenomes in the B genome were designated LF_B_, MF1_B_ and MF2_B_. Evolutionary relationship between At and the A and B genomes using a set of 1482 genes, present as three paralogs in each genome, showed LF_A_‐LF_B_, MF1_A_‐MF1_B_ and MF2_A_‐MF2_B_ to be the most related combinations (Table S20; Figure S15). The LF, MF1 and MF2 genomes diverged ~15MYA, homoeologs between A and B ~8.5 MYA and homoeologs between A and C ~4.5 MYA (Figure S16).

**Figure 2:**
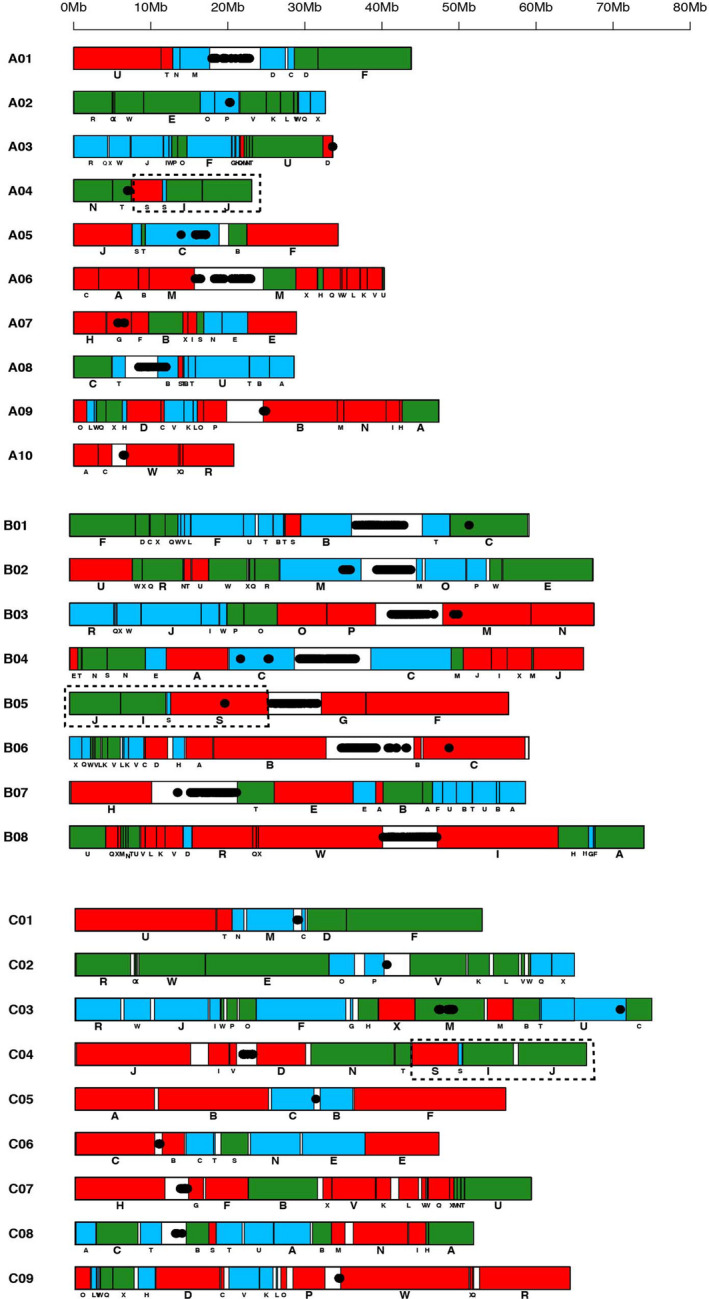
Gene block arrangements in the A and B genomes of *Brassica juncea* and C genome of *B. oleracea*. LF, MF1 and MF2 paleogenomes are represented by red, green and blue colours, respectively. A and C genomes show very similar block arrangements, whereas the B genome shows more fragmentation of the gene blocks.

**Figure 3:**
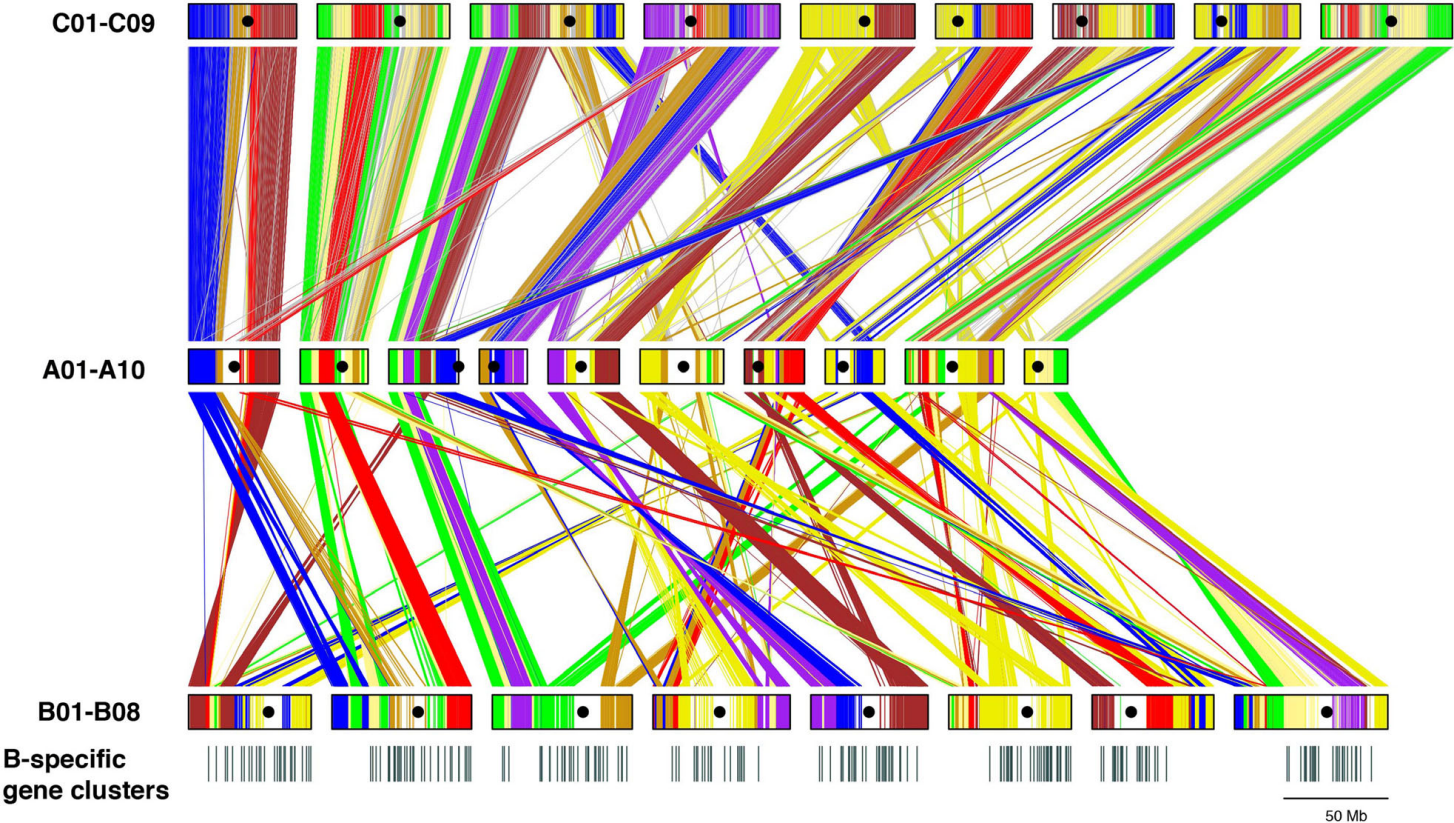
Segmental collinearity of the A and B genomes of *Brassica juncea* and C genome of *B. oleracea*. Gene blocks syntenous with *A. thaliana* are colour‐coded following Schranz *et al*. (2006). The A and C genomes have many similar gene block associations as compared to the A and B genomes. The B genome contains a very high number of genome‐specific genes many of which are present as clusters of ≥10 genes—such clusters have been represented as black bars below the B genome pseudochromosomes.

We assigned all the gene blocks represented in the A and B genomes of *B. juncea* and *B. oleracea* (CC) (Belser *et al*., 2018) to the three constituent paleogenomes—LF, MF1, and MF2 (Figure 2). The A and C genome homoeologs, besides being less divergent, show extensive similarity in their gene block arrangements as compared to the B genome homoeologs (Figure 3). Contiguous genome assemblies have shown the A, B and C genomes to be even more fragmented than reported earlier (Belser *et al*., 2018; Liu *et al*., 2014; Wang *et al*., 2011; Zhang *et al*., 2018) with extensive intra‐block fragmentation (Figure 2; Table S21). Gene block associations have been studied in the selected taxa of different tribes identified in the family *Brassicaceae* to understand inter‐tribe and intra‐tribe evolutionary relationships (Lysak *et al*., 2016) (Table S22). Given the more extensive gene block fragmentation observed in the A, B and C genomes, we tested the new structural information for any novel evolutionary insights. We scanned the gene block associations and classified these under four categories—(a) intra‐paleogenome contiguous gene block associations, (b) intra‐paleogenome non‐contiguous gene block associations, (c) inter‐paleogenome non‐contiguous gene block associations and (d) inter‐paleogenome same‐gene block associations (Table S23).

The ancestral intra‐paleogenome contiguous gene block associations—A‐B, F‐G, G‐H, T‐U and W‐X, reported to be absent in the A and C genomes of *B. rapa*, *B. oleracea* and *B. napus* are present in one or the other constituent paleogenomes (Figure 2; Table S23). The earlier assemblies missed these associations, and therefore, it was concluded that their absence was a characteristic feature of the tribe Brassicaceae (Lysak *et al*., 2016). Intra‐paleogenome non‐contiguous gene block associations are key to understand the pre‐***b*** event structure of the three paleogenomes. The previously reported association V‐K‐L‐W‐Q‐X does not exist in any of the Brassicaceae paleogenomes; only V‐K‐L is the shared association (Table S22 and 23). We report some new associations in this category—three of these Q‐X, W‐X‐Q‐R and V‐K‐L are present in all the three paleogenomes pointing to a shared ancestry; W‐E, M‐N‐T‐U and O‐P‐W are present only in MF1_A_, MF1_B_ and MF1_C_. Novel associations in the MF1 genome could predate the ***b*** event and could have contributed to gene block fragmentation upon hybridization. We looked at the inter‐paleogenome non‐contiguous gene block associations to find clues whether the A and B genomes resulted from independent ***b*** events. The presence of the gene block association J_MF1_‐I_MF1_‐S_MF2_‐S_LF_ with similar gene block junctions in all three genomes (Figure 2; Figure S17) shows that the A and B genomes resulted from a common ***b*** event.

## Discussion

We have reported a highly contiguous genome assembly of *B. juncea *variety Varuna, an oleiferous type belonging to the Indian gene pool of mustard, using long‐read SMRT sequencing and optical mapping. The *B. juncea* assembly reported here has given contig N50 value of >5Mb, comparable to the other recent SMRT based genome assemblies. We found recursive optical mapping with three different labelling reactions to be highly useful for correcting misassemblies. The final assembly showed a strong correlation with the genetic marker‐based maps, which were developed for this study. The Varuna assembly is a marked improvement over the previous assembly of *B. juncea* variety Tumida—particularly, for the B genome—providing a coverage of ~507.1 Mb as compared to a coverage of ~377.6 Mb in Tumida (V1.5).

We carried out a detailed comparison of the structural aspects of the BjuA and BjuB genomes with a recent assembly of *B. oleracea* (BolC) genome (Belsar *et al*., 2018). While the A and C genomes share many common structural features, the B genome seems to be disparate from the A and C genomes for the transposon content, centromeric repeats and gene block associations. We also compared the A genome of *B. juncea* (BjuA) with the recently updated *B. rapa* (BraA) V3.0 genome assembly (Zhang *et al*, 2018). The two A genomes have similar number of genes that show high collinearity in their arrangement. A comparison of the B genome of *B. juncea* and our recently assembled chromosome‐scale assembly of *B. nigra* Sangam has shown similar gene and transposable element content and high gene collinearity between the BjuB and BniB genomes (Paritosh *et al*., 2020). We can conclude that *B. juncea* is a strict allopolyploid of *B. rapa* and *B. nigra*. We did not observe any exchanges between the homoeologous regions of the BjuA and BjuB genomes. This might be due to a strong suppression of homoeologous pairing and high divergence in block arrangements between the two genomes.

While genome triplication (the ***b*** event) seems to be a characteristic feature of the genera belonging to the tribe Brassicaceae (Lysak and Koch, 2011), some intriguing questions remain—were structurally similar or dissimilar genomes involved in the ***b ***event, and was there a single or more than one independent hybridization event? We tested whether the contiguous assemblies of the A, B and C genomes available now would provide answers to the two questions posed above. The three intra‐paleogenome non‐contiguous gene block associations Q‐X, W‐X‐Q‐R and V‐K‐L are present in all the three paleogenomes—LF, MF1 and MF2 in the A, B and C genomes and might have been present in the genome ancestral to the three paleogenomes. Three intra‐paleogenome non‐contiguous gene block associations are present only in the MF1 genome of the A, B and C genomes. These novel associations could have been present in the MF1 genome prior to the ***b*** event.

Two different models have been proposed for the evolution of the Brassica genomes. One model suggested genome triplication with three near‐similar PCK (Proto‐Calepineae Karyotype; *n* = 7) genomes that precipitated differential gene loss, reduction in chromosome number, and new gene block associations followed by changes resulting from homoploid hybrid speciation (Cheng *et al*., 2017). Another model on genome triplication suggested that two of the three paleogenomes—MF1 and MF2, formed an allotetraploid with MF1 establishing dominance in gene retention, followed by crosses with the LF genome (the actual ***b*** event) leading to chromosomal restructuring and further gene fractionation (Tang *et al*., 2012). However, neither a single nor a two‐step ***b*** event resolves the issue of the presence of two distinct plastid lineages (the rapa lineage and the nigra lineage) in the tribe Brassiceae, which suggests more than one genome triplication event (Arias and Pires, 2012; Li *et al*., 2017; Pradhan *et al*., 1992; Warwick and Black, 1991). The two‐step model was developed further to accommodate the presence of the two distinct plastid genome lineages by suggesting reciprocal crosses between the tetraploid (MF1 + MF2) lineage and the LF lineage (Sharma *et al*, 2014). While the A and B genomes show very disparate gene block associations in general, the presence of a common inter‐paleogenome non‐contiguous gene block association J_MF1_–I_MF1_–S_MF2_–S_LF_ lends support to a monophyletic origin of the A and B genomes. A gene‐to‐gene collinearity analysis has shown similar junctions in the J_MF1_–I_MF1_–S_MF2_–S_LF_ of the A, B and C genomes.

We earlier identified two distinct gene pools in oilseed mustard—Indian and east European (Pradhan *et al*., 1993; Srivastava *et al*., 2001). Hybrids between the Indian and east European gene pool lines have been found to be heterotic for yield (Pradhan *et al*., 1993). The Indian gene pool lines like Varuna are well adapted to the dry land cultivation but require further breeding for yield increase and resistance to disease like white rust, Alternaria blight and stem rot. The east European lines, as such, are ill‐adapted to the growing conditions in south Asia due to photoperiod sensitivity but contain a large number of positive traits like high pod density (Ramchiary *et al*., 2007a,2007b; Yadava *et al*., 2012), oil and seed meal quality (Gupta *et al*., 2004; Rout *et al*., 2015), and disease resistance (Arora *et al*., 2019). Several QTL have been mapped in the Indian and east European gene pool lines (Dhaka *et al*., 2017; Rout *et al*., 2018). The Chinese vegetable types like Tumida constitute another divergent gene pool (Yang *et al*., 2016) that contains potentially useful traits like serrated leaves, basal branching (Muntha *et al*., 2018) and disease resistance (Bhayana *et al*., 2019). The highly contiguous genome sequence assembly of *B. juncea* Varuna will allow fine mapping in the QTL regions, identification of the candidate genes, and understanding the molecular mechanisms underlying some of the critical quantitative traits, thereby strengthening mustard breeding in south Asia and other parts of the world.

## Methods

### Plant material

*Brassica juncea* variety Varuna used for sequencing was maintained by strict selfing for over six generations. The other material used for sequencing— *B. nigra* (BB) variety Sangam (an Indian gene pool line), is used extensively for condiment purposes in India. As *B. nigra* is self‐incompatible and therefore highly heterozygous, sequencing was carried out on a derived doubled haploid (DH) line—named BnSDH‐1—which was maintained by selfing through bud pollination. A mapping population of *B. juncea* Varuna x Heera (VH population) was developed earlier (Panjabi *et al*., 2008; Pradhan *et al*., 2003). A new F_1_DH mapping population was developed in *B. juncea* from a Tumida × Varuna cross (TuV population). A *B. nigra* F_1_DH mapping population was developed from the cross—BnSDH‐1 × line 2782 (an exotic east European origin line). The DH mapping populations were maintained by strict self‐pollination in the field during the growing season.

### PacBio library construction and sequencing

For DNA isolation, *B. juncea* Varuna seedlings were grown in a growth chamber (light period 8 h, temp. 25 °C/dark period 16 h, temp. 10 °C). 15‐d‐old seedlings were kept in the dark for 24 h; young leaves were harvested and frozen immediately in liquid nitrogen. Leaf tissues were used to isolate the nuclear fraction; high molecular weight genomic DNA was extracted from the isolated nuclei and subjected to pulse‐field gel electrophoresis for separating DNA in the range of 30–50 kb for library preparation. Libraries were developed using ‘SMRTbell template preparation kit’ following the manufacturer’s instructions and sequenced on the PacBio RSII platform (https://www.pacb.com).

### Illumina and nanopore library construction and sequencing

*B. juncea* Varuna and *B. nigra* BnSHD‐1 plants were grown in a growth chamber under conditions described above and used for DNA isolation by CTAB method (Rogers and Bendich, 1994). DNA was purified using the ‘DNase Mini kit’ (Qiagen). Approximately 5 µg of genomic DNA was fragmented with a focused ultrasonicator system (Covaris). Fragmented DNA was used to prepare Illumina paired‐end (PE) libraries of size 200–350 bp for *B. juncea* Varuna following the manufacturer’s recommended protocol (https://illumina.com). For *B. nigra,* three different PE libraries of sizes 200–350 bp, 300–450 bp, and 400–550 bp and three mate‐pair (MP) libraries with the size range of 2–3 kb, 4–6 kb, and 10 kb were constructed as per the recommended protocols. The PE libraries were sequenced on Illumina HiSeq 1000 sequencer and MP libraries on the Illumina MiSeq system. For Oxford Nanopore technology (https://nanoporetech.com) based genome sequencing of *B. nigra*, around 3 µg of purified high molecular weight DNA was used directly for developing sequencing libraries following ‘1D gDNA selection for long reads’ protocol using the ‘Ligation sequencing kit 1D’ recommended by the manufacturer ((https://nanoporetech.com). Developed libraries were sequenced with R9.4 SpotON Minion‐106 Flow cells. Base‐calling was performed upon completion of the sequencing runs with Albacore software (https://github.com/Albacore).

### Assembly of *B. juncea* and *B. nigra* genomes

Sequencing of *B. juncea* libraries on the PacBio Sequel platform generated around 9 million long reads; these were processed by Canu assembler (V1.4) (Koren *et al*., 2017) with ‘minReadLength’ and ‘minOverlapLength’ set at 1000 bp, ‘rawErrorRate’ set at 0.3, and ‘correctedErrorRate’ at 0.045. Completeness of the assembly was checked by mapping sequences obtained from the Illumina PE libraries on the assembled SMRT based contigs using BWA mem (Li and Durbin, 2009) set at default parameters.

Sequences generated from the three PE libraries of *B. nigra* were assembled into contigs with MaSuRcA software (Zimin *et al*., 2013). Illumina PE reads were mapped on the raw Nanopore reads using BWA mem; 1 442 476 error corrected Nanopore reads were generated using the Pilon program (Walker *et al*., 2014). Scaffolding of the MaSuRcA based contigs with the error corrected Nanopore sequence data was carried out with SSPACE‐LongRead.pl script (Boetzer *et al*., 2011), which generated 29 808 scaffolds with an N50 value of 19 834. Further, three rounds of scaffolding were carried out using the 2 kb, 6 kb and 10 kb MP library sequence data sets using SSPACE‐STANDARD‐3.0.pl script (Boetzer *et al*., 2011).

### Optical mapping, scaffolding and error correction

BioNano optical maps were developed with proprietary kits and protocols provided by BioNano Genomics (https://bionanogenomics.com). High molecular weight DNA was isolated from 1g tissue of freshly harvested etiolated young leaves of *B. juncea* using ‘IrysPrep Plant Tissue DNA isolation kit’. Approximately 750 ng of the genomic DNA was trapped in agarose gel plugs and labelled at nicks introduced by *BssS*I and *BspQ*I enzymes in separate reactions using the ‘IrysPrep NLRS labeling kit’. Around 900 ng of high molecular weight DNA was used for DLS labelling using ‘DNA labeling kit‐DLS’. For each reaction, labelled DNA was imaged on the Saphyr system (https://bionanogenomics.com) using three lanes of the Saphyr Chip. For each of the three libraries, pairwise comparison was performed with RefAligner to identify all molecule overlaps, and three different consensus maps were constructed. All fragments were then mapped back to the consensus maps which were recursively refined and extended. The BioNano IrysSolve module ‘HybridScaffold’ was used to perform the hybrid assembly between PacBio‐SMRT contigs and BioNano‐assembled genome maps. The scaffolds and unscaffolded contigs were polished by the Illumina PE reads using Pilon software (Walker *et al*., 2014).

### Genetic mapping

Marker‐based genetic maps were developed from two F_1_DH populations of *B. juncea*—Varuna x Heera (VH) and Tumida x Varuna (TuV). VH population was developed earlier and contained 709—intron polymorphic (IP), genic SSR and SNP markers (Panjabi *et al*., 2008). A subset of 92 F_1_DH lines of the VH population along with the two parents was used for GBS (genotyping by sequencing) based mapping using *Sph*I‐*Mluc*I restriction enzyme combination (He *et al*., 2014). PE reads that were generated from the sequencing of the F_1_DH individuals, and the two parental lines were mapped to the scaffolds and contigs generated with PacBio‐SMRT sequencing and Optical mapping using BWA. SNPs were identified, and potential markers were filtered—markers with read‐depth <10, monomorphic markers and markers with >30 missing data in the population were rejected. Genetic mapping was carried out in the R statistical computing environment using the ASMap package (Taylor and Butler, 2017). The R/ASMap package uses an R/qtl (Broman *et al*., 2003) format for the structure of its genetic objects and utilizes the MSTmap algorithm (Wu *et al*., 2008). Marker data were assembled to be used as a DH population type and transformed into the R/qtl format. The genetic map was calculated by using the mstmap function with the default parameters: distance function Kosambi, cut‐off p‐value 1e‐10 and missing threshold 0.3. GBS marker tags of the VH population were used to anchor the assembled super‐scaffolds and contigs on the genetic map. Anchored scaffolds and contigs were stitched into 18 pseudochromosomes using custom Perl scripts. Correlations were drawn between the GBS markers on the genetic map and their physical position on the pseudochromosomes using custom R scripts.

For the genetic map of the TuV population, a total of 119 lines were used—first for genotyping with 524 anchor markers (179 IP, 184 SNP and 161 SSR) that were common with the VH population. Subsequently, 8517 GBS markers were mapped using the same parameters as used for the VH genetic map, except that the DNA was restricted with the *Hinf*I‐*Hpy*CH*4*IV enzyme combination that is reported to provide more extensive genome coverage (Fu *et al*., 2016). PE reads that were generated from the sequencing of the F_1_DH individuals and the two parental lines were mapped to the 18 pseudochromosomes and the scaffolds and contigs that remained unassigned after analysis with the VH genetic markers using BWA. GBS marker filtering, genetic mapping and correlations in the form of dot plots were drawn between the physical positions of the GBS based SNPs and their genetic locations on the TuV genetic map as done for the VH map.

For genetic mapping in *B. nigra*, 92 F_1_DH lines derived from the cross Sangam × 2782 were used; a framework genetic map was developed with 208 anchor markers (180 IP and 28 SSR) that were common with the VH anchor map. Further, 2723 GBS markers (enzyme combination *Sph*I‐*Mluc*I) were added using the mapping procedures described above. *B. nigra* pseudochromosomes were developed by organizing the assembled scaffolds as per the order of the markers on the genetic map.

### Transcriptome sequencing

For *B. juncea*, PacBio based transcriptome sequencing (Iso‐seq) was carried out on total RNA isolated from a pooled sample of seedlings, leaves, inflorescence with developing siliqua along with the seeds using ‘Spectrum plant total RNA kit’ (Sigma). The quality of RNA was checked on Bioanalyzer 2100 (Agilent) using the ‘RNA 6000 Nano kit’. 5 µg of high‐quality RNA (RIN value ≥ 7) was used for library preparation using the ‘Iso‐Seq SMRTBell library preparation kit’. Two size ranges were collected, one of size range 0.5‐1kb and the other of 1–2 kb. Raw sequencing reads for each of the libraries were generated using P4‐C6 chemistry on the PacBio RS II sequencing machine. Sequencing data were generated from seven SMRT cells for ≤1 kb library and eight SMRT cells for 1–2 kb library. Sequences were assembled separately using SMRT Link software (https://www.pacb.com/). For validating the genes predicted in the Varuna genome, besides the Iso‐seq data—different transcriptome data sets deposited in the NCBI SRA database (https://www.ncbi.nlm.nih.gov/sra) were downloaded and used. QC was carried out using Trimmomatic software (Bolger *et al*., 2014) and mapped on the assembled genome using STAR aligner (Dobin *et al*., 2013).

For the transcriptome assembly of *B. nigra*, RNA was isolated from the seedlings, leaves and developing inflorescence tissues. 6 μg of RNA (RIN value ≥ 7) was used for cDNA library preparation following the method described earlier (Paritosh *et al*., 2013). PE library with an insert size range of 250–370 bp was developed and sequenced (2 × 100 bp) using Genome Analyzer IIx sequencer (Illumina). Quality filtering and the raw reads were carried out with Trimmomatic (Bolger *et al*., 2014) and Fastx‐Toolkit (http://hannonlab.cshl.edu/fastx_toolkit/). Sequences with Phred value < 20 in 70% of the bases of the sequence were discarded. Filtered paired‐end sequences were assembled with Trinity software (Grabherr *et al*., 2011). Transcriptome sequenced on GS FLX Titanium platform (Roche) was assembled with Newbler software (Papanicolaou *et al*., 2009).

### Gene and transposable element prediction

Transposable elements (TEs) were defined into different subgroups based on criteria specified earlier (Wicker *et al*., 2007). For TE predictions, a *de novo* library was constructed using the RepeatModeler pipeline (http://www.repeatmasker.org/RepeatModeler/). The initial repeat database was generated using RECON (Bao and Eddy, 2002), RepeatScout (Price *et al*., 2005), Tandem Repeat Finder (Benson and Dong, 1999) and NSEG (ftp://ftp.ncbi.nih.gov/pub/seg/nseg/) programs. LTRs were further identified using LTRfinder (Xu and Wang, 2007) program. A reference library for the consensus repeats was developed and merged with the known repbase database of *A. thaliana*. This TE library was used to predict TE in the assembled genome using RepeatMasker (http://www.repeatmasker.org).

The repeat‐masked DNA sequences were used to annotate the protein‐coding genes using Augustus program (Stanke and Morgenstern, 2005). A total of 543 randomly selected full‐length CDS sequences were used as a training data set for Augustus *ab‐initio* prediction. The predicted genes were further validated by the UniProt database (https://uniprot.org). Genes with <50 bp coding length and TE‐related genes were removed. Predicted genes were validated by mapping these with previous transcriptome analysis data sets of *B. juncea* using STAR aligner. Further, Iso‐seq data were used to validate the predicted genes and to find the alternative transcript isoforms. Iso‐seq data were mapped on the assembled pseudochromosomes with Minimap2 program (Li, 2018).

### Centromere region‐specific repeats

Potential centromeric regions were identified from the correlation plots between GBS markers on the genetic maps and physical position of their respective tags on the pseudochromosomes. Python‐based scripts were developed to identify kmers of 100bp length with >20 times occurrence in the predicted centromeric regions. The identified kmers were assembled using DNAstar (https://www.dnastar.com/) to get consensus contigs. The assembled contigs were further analysed for their presence in the genome assembly by Blast search analysis, and centromere‐specific sequences were selected for both A and B genomes, separately.

### Syntenic block construction and determination of gene fractionation patterns

Syntenic gene blocks were constructed with MCScanX (Wang *et al*., 2012) program. The all‐against‐all Blastp comparison was carried out against A and B genomes of *B. juncea* with At. MCScanX was run with the default parameters—match_score: 50, match_size: 5, gap_penalty: −1, e‐value: 1e‐05 and max_gaps: 25. The output was validated with the SynFind program at the CoGE website (https://genomevolution.org/coge). At least three syntenic regions were identified both in the A and B genomes for each gene block of *A. thaliana*. Gene retention pattern for each of the syntenic regions was calculated as a proportion of genes retained for 500 flanking genes around a given gene locus considering the *A. thaliana* syntenic region as 100% retained. Based on the gene retention pattern of the syntenic regions, the gene blocks were grouped into LF, MF1 and MF2 as defined in *B. rapa* (Wang *et al*., 2011). The SynFind program at the CoGE website was also used to find out any significant homoeologous exchanges (≥5 consecutive genes) between the A and B subgenomes of *B. juncea*.

For orthologue identification of the B genome‐specific genes, a two‐step gene identification pipeline was implemented. In the first step, protein sequences from A and B genomes of *B. juncea* were compared to *B. oleracea* (C genome) and At using OrthoFinder software (Emms and Kelly, 2015). All‐against‐all sequence comparison was carried out using Diamond software (Buchfink *et al*., 2015).

### Phylogenetic analysis of gene families

1482 genes of At that were present as a single‐copy gene in At (representing all the ancestral gene blocks; A–X) and three orthologues each in the A, B and C genomes were selected for phylogenetic analysis. The amino acid sequences of orthologues were aligned using MUSCLE (v3.8.31) (Edgar, 2004). Poorly aligned regions were trimmed using GBLOCKS (v0.91, Talavera and Castresana, 2007) and subsequently with PAL2NAL script (Suyama *et al*., 2006). Concatenation of the sequences and conversion to Phylip format was performed using in‐house developed Perl scripts. Newick format trees were developed; Ka/Ks, and omega values were obtained using the PAML package (Yang, 2007). Divergence time was calculated assuming a mutation rate of 1.5 × 10^−8^ substitutions per synonymous site per year (Koch *et al*., 2000).

Significance of variation between mean Ks values for orthologue divergence (AT–A, AT–B and AT–C genomes), paralog divergence (within A, B and C genomes) and homoeolog divergence (between A, B and C genomes) was analysed statistically by one‐way ANOVA and Tukey’s post hoc test; *P* < 0.05 was considered to be statistically significant.

## Competing interests

The authors declare no competing interests.

## Author contributions

KP, JZ, DAK, DC and RAW carried out the genome assembly; VBRL generated optical maps; KP and NCB carried out transcriptome analysis; SKY, KP, PS, LB and VG did the mapping work; AM developed DH lines; KP did gene annotation and the other bioinformatics analysis; KP and DP wrote the manuscript; and RAW, AKP and DP supervised the study. All the authors read and approved the final manuscript.

## Code availability

Codes used in the manuscript are available on request.

## Supporting information


**Figure S1.** Genetic map of *Brassica juncea* Varuna x Heera (VH) F_1_DH population.
**Figure S2.** Genetic map of *Brassica juncea* Tumida x Varuna (TuV) F_1_DH population.
**Figure S3.** Genetic map of *Brassica nigra* Sangam x 2782 F_1_DH population.
**Figure S4.** Relationship between the GBS markers on the genetic map of *Brassica nigra* Sangam x 2782 F_1_DH population and physical position of the respective marker tags on the *B. nigra* genomic sequences.
**Figure S5.** Workflow in BioNano optical mapping based hierarchical scaffolding analysis of *Brassica juncea* genome.
**Figure S6.** Relationship between the GBS markers on the *Brassica juncea* VH (Varuna x Heera) F1DH population linkage map, and physical position of the respective marker tags on the assembled Varuna genome.
**Figure S7.** Relationship between the GBS markers on the *Brassica juncea* Tumida x Varuna F_1_DH population genetic map and the physical position of the respective GBS markers on the assembled Varuna genome.
**Figure S8.** Distribution of different types of transposable elements on *Brassica juncea* pseudochromosomes.
**Figure S9.** Position of the predicted A genome centromeric sequences on different pseudochromosomes of the A genome of *Brassica juncea*.
**Figure S10.** Position of the predicted B genome centromeric sequences on different pseudochromosomes of the B genome of *Brassica juncea*.
**Figure S11a.** Comparison of the genome assembly of *Brassica juncea* Varuna with previous assemblies of *B. rapa* Chiifu for the A genome and *B. juncea* Tumida for both the A and B genomes.
**Figure S11b.** Comparative gene‐to‐gene based collinearity analysis of the *Brassica jucnea* Varuna genome with *B. rapa* Chiifu V3.0 assembly and *B. nigra* Sangam long‐read assembly.
**Figure S12.** Gene density on *Brassica juncea* A genome pseudochromosomes.
**Figure S13.** Gene density on *Brassica juncea* B genome pseudochromosomes.
**Figure S14.** Proportion of orthologous genes retained in the A and B genomes of *Brassica juncea*.
**Figure S15.** Relationship of the three paleogenomes (LF, MF1, and MF2) of the A and B genomes of *Brassica juncea* based on Ks values.
**Figure S16.** Neighbour‐joining tree based divergence analysis of the constituent paleogenomes of the A, B, and C genomes as compared to *A. thaliana*.
**Figure S17.** Gene collinearity and junction analysis of the gene block association J_MF1_‐I_MF1_‐S_MF2_‐S_LF_ in the BjuA, BjuB, and BolC genomes.

**Table S1.** *Brassica juncea* variety Varuna raw sequencing data obtained with PacBio RSII platform.
**Table S2**. Statistics of paired‐end (PE) reads obtained with Illumina based sequencing of *B. juncea* variety Varuna.
**Table S3**. *Brassica nigra* variety Sangam sequencing data obtained with (a) Illumina paired‐end and (b) mate‐pair libraries, and (c) Nanopore sequencing; raw data‐filtering, trimming and quality statistics.
**Table S4**. Genome assembly statistics of *Brassica nigra* variety Sangam.
**Table S5**. Characteristics of the linkage map of *Brassica juncea* developed using Varuna x Heera (VH) F_1_DH mapping population.

**Table S6**. Description of the components of the *Brassica juncea* VH (Varuna x Heera) genetic map.
**Table S7**. Characteristics of the linkage map of *Brassica juncea* developed using Tumida × Varuna (TuV) F_1_DH mapping population.

**Table S8.** Description of the components of the *Brassica juncea* TuV (Tumida x Varuna) genetic map.
**Table S9**. Features of the linkage map of *Brassica nigra* developed using Sangam x IC2782 F_1_DH mapping population.

**Table S10.** Description of the components of the *Brassica nigra* Sangam x IC2782 genetic map.
**Table S11** **a**. Mapping data generated at each step of the hierarchical optical mapping analysis of *Brassica juncea* Varuna genome.
**Table S11** **b**. Statistics of the *Brassica juncea* genome after each round of Pilon correction.
**Table S12**. Position and number of the assembled scaffolds and contigs on each of the eighteen pseudochromosomes of *Brassica juncea*.
**Table S13**. A comparison of the features of the assembled *B. juncea* Varuna genome with previously reported genome of *Brassica juncea* Tumida (V1.1 and V1.5).
**Table S14**. *Brassica juncea* Varuna genome—distribution of TEs and other repeats on the A and B genomes.
**Table S15**. *Brassica juncea* Varuna genome—centromere‐specific repeat sequences identified in the A genome.
**Table S16**. *Brassica juncea* Varuna genome—centromere‐specific repeat sequences identified in the B genome.
**Table S17**. *Brassica juncea* Varuna genome—centromere‐specific transposable elements in the B genome.

**Table S18**. A catalogue of the genes predicted on different pseudochromosomes of *Brassica juncea* Varuna and their orthologues in *Arabidopsis thaliana* (along with their respective gene blocks) and *B. rapa* Chiifu, and homoeologs in the A or the B genome of *B. juncea*.
**Table S19**. Blockwise number of genes retained in the three constituent paleogenomes of the A and B genomes of *Brassica juncea*.
**Table S20**. Divergence analysis between *A. thaliana* and the constituent paleogenomes of the A and B genomes of *Brassica juncea*, based on synonymous nucleotide substitutions in genes.
**Table S21**. Genomic block fragmentation patterns in the A and B genomes of *Brassica juncea* Varuna.
**Table S22**. Ancient gene block associations described in the published studies as summarized by Lysak et al. (2016). The gene block associations in the A and B genomes of *Brassica juncea* reported in this study and C genome of *B. oleracea* using the assembly of Belsar et al (2018).
**Table S23**. Gene block associations between the ancestral paleogenomes that constituted the A, B genomes of *B. juncea,* and the C genome of *B. oleracea*. Red, green and blue colour represents the LF, MF1 and MF2 paleogenomes, respectively.

**File S1.** Transcriptome assembly of *Brassica juncea* Varuna and *Brassica nigra* Sangam.

## Data Availability

The genome sequence reads of *B. juncea* variety Varuna have been deposited under NCBI bioproject PRJNA550308. *B. nigra* genome and transcriptome sequences have been deposited under NCBI bioproject PRJNA324621. The *B. juncea* transcriptome data, generated with PacBio sequencing, has been deposited under NCBI bioproject PRJNA245462. Genome assembly can be downloaded from the website ‘http://cgmcp.du.ac.in/’ and is also available at the NCBI database under accession number JADMCG000000000.

## References

[pbi13492-bib-0001] Arias, T. and Pires, J.C. (2012) A fully resolved chloroplast phylogeny of the Brassica crops and wild relatives (Brassicaceae: Brassiceae): Novel clades and potential taxonomic implications. Taxon, 61, 980–988.

[pbi13492-bib-0002] Arora, H. , Padmaja, K.L. , Paritosh, K. , Mukhi, N. , Tewari, A.K. , Mukhopadhyay, A. , Gupta, V. *et al*. (2019) BjuWRR1, a CC‐NB‐LRR gene identified in *Brassica juncea*, confers resistance to white rust caused by *Albugo candida* . Theor. Appl. Genet. 132, 2223–2236. 31049632 10.1007/s00122-019-03350-z

[pbi13492-bib-0003] Bao, Z.R. and Eddy, S.R. (2002) Automated *de novo* identification of repeat sequence families in sequenced genomes. Genome Res. 12, 1269–1276. 12176934 10.1101/gr.88502 PMC186642

[pbi13492-bib-0004] Belser, C. , Istace, B. , Denis, E. , Dubarry, M. , Baurens, F.C. , Falentin, C. *et al*. (2018) Chromosome‐scale assemblies of plant genomes using nanopore long reads and optical maps. Nat. Plants, 4, 879–887. 30390080 10.1038/s41477-018-0289-4

[pbi13492-bib-0005] Benson, G. and Dong, L. (1999) Reconstructing the duplication history of a tandem repeat. In Proceedings of the Seventh International Conference on Intelligent Systems for Molecular Biology ( Lengauer, T. , Schneider, R. , Bork, P. , Brutlag, D. , Glasgow, J. , Mewes, H.W. , and Zimmer R. , eds), pp. 44–53. California: AAAI Press. 10786285

[pbi13492-bib-0006] Bhardwaj, A.R. , Joshi, G. , Kukreja, B. , Malik, V. , Arora, P. , Pandey, R. *et al*. (2015) Global insights into high temperature and drought stress regulated genes by RNA‐Seq in economically important oilseed crop *Brassica juncea* . BMC Plant Biol. 15, 9. 25604693 10.1186/s12870-014-0405-1 PMC4310166

[pbi13492-bib-0007] Bhayana, L. , Paritosh, K. , Arora, H. , Yadava, S.K. , Singh, P. , Nandan, D. *et al*. (2019) A Mapped locus on LG A6 of *Brassica juncea* line Tumida conferring resistance to white rust contains a CNL Type R Gene. Front. Plant Sci. 10, 1690. 31998351 10.3389/fpls.2019.01690 PMC6960627

[pbi13492-bib-0008] Boetzer, M. , Henkel, C.V. , Jansen, H.J. , Butler, D. and Pirovano, W. (2011) Scaffolding pre‐assembled contigs using SSPACE. Bioinformatics, 27, 578–579. 21149342 10.1093/bioinformatics/btq683

[pbi13492-bib-0009] Bolger, A.M. , Lohse, M. and Usadel, B. (2014) Trimmomatic: a flexible trimmer for Illumina sequence data. Bioinformatics, 30, 2114–2120. 24695404 10.1093/bioinformatics/btu170 PMC4103590

[pbi13492-bib-0010] Broman, K.W. , Wu, H. , Sen, S. and Churchill, G.A. (2003) R/qtl: QTL mapping in experimental crosses. Bioinformatics, 19, 889–890. 12724300 10.1093/bioinformatics/btg112

[pbi13492-bib-0011] Buchfink, B. , Xie, C. and Huson, D.H. (2015) Fast and sensitive protein alignment using DIAMOND. Nat. Methods, 12, 59–60. 25402007 10.1038/nmeth.3176

[pbi13492-bib-0012] Chalhoub, B. , Denoeud, F. , Liu, S. , Parkin, I.A. , Tang, H. *et al*. (2014) Early allopolyploid evolution in the post‐Neolithic *Brassica napus* oilseed genome. Science, 345, 950–953. 25146293 10.1126/science.1253435

[pbi13492-bib-0013] Chauhan, J. , Singh, K. and Kumar, S. (2011) Hundred years of rapeseed‐mustard breeding in India: Accomplishments and future strategies. Indian J. Agri. Sci. 81, 1093–1109.

[pbi13492-bib-0014] Cheng, F. , Liang, J. , Cai, C. , Cai, X. , Wu, J. and Wang, X. (2017) Genome sequencing supports a multi‐vertex model for Brassiceae species. Curr. Opin. Plant Biol. 36, 79–87. 28242534 10.1016/j.pbi.2017.01.006

[pbi13492-bib-0015] Dhaka, N. , Rout, K. , Yadava, S.K. , Sodhi, Y.S. , Gupta, V. , Pental, D. and Pradhan, A.K. (2017) Genetic dissection of seed weight by QTL analysis and detection of allelic variation in Indian and east European gene pool lines of *Brassica juncea* . Theor. Appl. Genet. 130, 293–307. 27744489 10.1007/s00122-016-2811-2

[pbi13492-bib-0016] Dobin, A. , Davis, C.A. , Schlesinger, F. , Drenkow, J. , Zaleski, C. , Jha, S. *et al*. (2013) STAR: ultrafast universal RNA‐seq aligner. Bioinformatics, 29, 15–21. 23104886 10.1093/bioinformatics/bts635 PMC3530905

[pbi13492-bib-0017] Edgar, R.C. (2004) MUSCLE: a multiple sequence alignment method with reduced time and space complexity. BMC Bioinformat. 5, 113. 10.1186/1471-2105-5-113 PMC517706 15318951

[pbi13492-bib-0018] Emms, D.M. and Kelly, S. (2015) OrthoFinder: solving fundamental biases in whole genome comparisons dramatically improves orthogroup inference accuracy. Genome Biol. 16, 157. 26243257 10.1186/s13059-015-0721-2 PMC4531804

[pbi13492-bib-0019] Fu, Y.B. , Peterson, G.W. and Dong, Y. (2016) Increasing genome sampling and improving SNP genotyping for genotyping‐by‐sequencing with new combinations of restriction enzymes. G3, 6, 845–856. 26818077 10.1534/g3.115.025775 PMC4825655

[pbi13492-bib-0020] Grabherr, M.G. , Haas, B.J. , Yassour, M. , Levin, J.Z. , Thompson, D.A. , Amit, I. *et al*. (2011) Full‐length transcriptome assembly from RNA‐Seq data without a reference genome. Nat. Biotechnol. 29, 644–652. 21572440 10.1038/nbt.1883 PMC3571712

[pbi13492-bib-0021] Gupta, V. , Mukhopadhyay, A. , Arumugam, N. , Sodhi, Y.S. , Pental, D. and Pradhan, A.K. (2004) Molecular tagging of erucic acid trait in oilseed mustard (*Brassica juncea*) by QTL mapping and single nucleotide polymorphisms in *FAE1* gene. Theor. Appl. Genet. 108, 743–749. 14564400 10.1007/s00122-003-1481-z

[pbi13492-bib-0022] He, J. , Zhao, X. , Laroche, A. , Lu, Z.X. , Liu, H. and Li, Z. (2014) Genotyping‐by‐sequencing (GBS), an ultimate marker‐assisted selection (MAS) tool to accelerate plant breeding. Front. Plant Sci. 5, 484. 25324846 10.3389/fpls.2014.00484 PMC4179701

[pbi13492-bib-0023] Jat, R.S. , Singh, V.V. , Sharma, P. and Rai, P.K. (2019) Oilseed Brassica in India: Demand, supply, policy perspective and future potential. OCL, 26, 8.

[pbi13492-bib-0024] Koch, M.A. , Haubold, B. and Mitchell‐Olds, T. (2000) Comparative evolutionary analysis of chalcone synthase and alcohol dehydrogenase loci in *Arabidopsis*, *Arabis*, and related genera (*Brassicaceae*). Mol. Biol. Evol. 17, 1483–1498. 11018155 10.1093/oxfordjournals.molbev.a026248

[pbi13492-bib-0025] Koren, S. , Walenz, B.P. , Berlin, K. , Miller, J.R. , Bergman, N.H. and Phillippy, A.M. (2017) Canu: scalable and accurate long‐read assembly via adaptive k‐mer weighting and repeat separation. Genome Res. 27, 722–736. 28298431 10.1101/gr.215087.116 PMC5411767

[pbi13492-bib-0026] Li, H. (2018) Minimap2: pairwise alignment for nucleotide sequences. Bioinformatics, 34, 3094–3100. 29750242 10.1093/bioinformatics/bty191 PMC6137996

[pbi13492-bib-0027] Li, H. and Durbin, R. (2009) Fast and accurate short read alignment with Burrows‐Wheeler transform. Bioinformatics, 25, 1754–1760. 19451168 10.1093/bioinformatics/btp324 PMC2705234

[pbi13492-bib-0028] Li, C.S. , Lin, F. , An, D. , Wang, W.Q. and Huang, R.D. (2018) Genome sequencing and assembly by long reads in plants. Genes, 9, 6. 10.3390/genes9010006 PMC5793159 29283420

[pbi13492-bib-0029] Li, P. , Zhang, S. , Li, F. , Zhang, S. , Zhang, H. , Wang, X. *et al*. (2017) A phylogenetic analysis of chloroplast genomes elucidates the relationships of the six economically important Brassica species comprising the triangle of U. Front. Plant Sci. 8, 111. 28210266 10.3389/fpls.2017.00111 PMC5288352

[pbi13492-bib-0030] Liu, S. , Liu, Y. , Yang, X. , Tong, C. , Edwards, D. , Parkin, I.A. *et al*. (2014) The *Brassica oleracea* genome reveals the asymmetrical evolution of polyploid genomes. Nat. Commun. 5, 3930. 24852848 10.1038/ncomms4930 PMC4279128

[pbi13492-bib-0031] Lysak, M.A. and Koch, M.A. (2011) Phylogeny, Genome, and Karyotype Evolution of Crucifers (Brassicaceae). In Genetics and Genomics of the Brassicaceae( Schmidt, R. and Bancroft, I. , eds), pp. 1–31. New York, NY: Springer New York.

[pbi13492-bib-0032] Lysak, M.A. , Koch, M.A. , Pecinka, A. and Schubert, I. (2005) Chromosome triplication found across the tribe Brassiceae. Genome Res. 15, 516–525. 15781573 10.1101/gr.3531105 PMC1074366

[pbi13492-bib-0033] Lysak, M.A. , Mandakova, T. and Schranz, M.E. (2016) Comparative paleogenomics of crucifers: ancestral genomic blocks revisited. Curr. Opin. Plant Biol. 30, 108–115. 26945766 10.1016/j.pbi.2016.02.001

[pbi13492-bib-0034] Muntha, S.T. , Zhang, L. , Zhou, Y. , Zhao, X. , Hu, Z. , Yang, J. and Zhang, M. (2018) Phytochrome A signal transduction 1 and CONSTANS‐LIKE 13 coordinately orchestrate shoot branching and flowering in leafy *Brassica juncea* . Plant Biotechnol. J. 17, 1333–1343. 10.1111/pbi.13057 PMC6576096 30578711

[pbi13492-bib-0059] Nagaharu, U. (1935) Genome analysis of Brassica with special reference to the experimental formation of *B. napus* and peculiar mode of fertilization. Japan J. Bot. 7, 389–452.

[pbi13492-bib-0035] Panjabi, P. , Jagannath, A. , Bisht, N.C. , Padmaja, K.L. , Sharma, S. , Gupta, V. *et al*. (2008) Comparative mapping of *Brassica juncea* and *Arabidopsis thaliana* using Intron Polymorphism (IP) markers: homoeologous relationships, diversification and evolution of the A, B and C Brassica genomes. BMC Genom. 9, 113. 10.1186/1471-2164-9-113 PMC2277410 18315867

[pbi13492-bib-0036] Papanicolaou, A. , Stierli, R. , Ffrench‐Constant, R.H. and Heckel, D.G. (2009) Next generation transcriptomes for next generation genomes using est2assembly. BMC Bioinform. 10, 447. 10.1186/1471-2105-10-447 PMC3087352 20034392

[pbi13492-bib-0037] Paritosh, K. , Gupta, V. , Yadava, S.K. , Singh, P. , Pradhan, A.K. and Pental, D. (2014) RNA‐seq based SNPs for mapping in *Brassica juncea* (AABB): synteny analysis between the two constituent genomes A (from *B. rapa*) and B (from *B. nigra*) shows highly divergent gene block arrangement and unique block fragmentation patterns. BMC Genom. 15, 396. 10.1186/1471-2164-15-396 PMC4045973 24886001

[pbi13492-bib-0038] Paritosh, K. , Pradhan, A.K. , and Pental, D. (2020) A highly contiguous genome assembly of Brassica nigra (BB) and revised nomenclature for the pseudochromosomes. bioRxiv, doi: 10.1101/2020.06.29.175869. PMC7731534 33308149

[pbi13492-bib-0039] Paritosh, K. , Yadava, S.K. , Gupta, V. , Panjabi‐Massand, P. , Sodhi, Y.S. , Pradhan, A.K. and Pental, D. (2013) RNA‐seq based SNPs in some agronomically important oleiferous lines of *Brassica rapa* and their use for genome‐wide linkage mapping and specific‐region fine mapping. BMC Genom. 14, 463. 10.1186/1471-2164-14-463 PMC3711843 23837684

[pbi13492-bib-0040] Parkin, I.A. , Gulden, S.M. , Sharpe, A.G. , Lukens, L. , Trick, M. , Osborn, T.C. and Lydiate, D.J. (2005) Segmental structure of the *Brassica napus* genome based on comparative analysis with *Arabidopsis thaliana* . Genetics, 171, 765–781. 16020789 10.1534/genetics.105.042093 PMC1456786

[pbi13492-bib-0041] Pradhan, A.K. , Gupta, V. , Mukhopadhyay, A. , Arumugam, N. , Sodhi, Y.S. and Pental, D. (2003) A high‐density linkage map in *Brassica juncea* (Indian mustard) using AFLP and RFLP markers. Theor. Appl. Genet. 106, 607–614. 12595988 10.1007/s00122-002-1083-1

[pbi13492-bib-0042] Pradhan, A.K. , Prakash, S. , Mukhopadhyay, A. and Pental, D. (1992) Phylogeny of Brassica and allied genera based on variation in chloroplast and mitochondrial DNA patterns: molecular and taxonomic classifications are incongruous. Theor. Appl. Genet. 85, 331–340. 24197323 10.1007/BF00222878

[pbi13492-bib-0043] Pradhan, A.K. , Sodhi, Y.S. , Mukhopadhyay, A. and Pental, D. (1993) Heterosis breeding in Indian mustard (Brassica‐Juncea L Czern and Coss) ‐ analysis of component characters contributing to heterosis for yield. Euphytica, 69, 219–229.

[pbi13492-bib-0044] Price, A.L. , Jones, N.C. and Pevzner, P.A. (2005) *De novo* identification of repeat families in large genomes. Bioinformatics, 21, i351–i358. 15961478 10.1093/bioinformatics/bti1018

[pbi13492-bib-0045] Ramchiary, N. , Bisht, N.C. , Gupta, V. , Mukhopadhyay, A. , Arumugam, N. , Sodhi, Y.S. *et al*. (2007a) QTL analysis reveals context‐dependent loci for seed glucosinolate trait in the oilseed *Brassica juncea*: importance of recurrent selection backcross scheme for the identification of 'true' QTL. Theor. Appl. Genet. 116, 77–85. 17898985 10.1007/s00122-007-0648-4

[pbi13492-bib-0046] Ramchiary, N. , Padmaja, K.L. , Sharma, S. , Gupta, V. , Sodhi, Y.S. , Mukhopadhyay, A. *et al*. (2007b) Mapping of yield influencing QTL in *Brassica juncea*: implications for breeding of a major oilseed crop of dryland areas. Theor. Appl. Genet. 115, 807–817. 17646960 10.1007/s00122-007-0610-5

[pbi13492-bib-0047] Rogers, S.O. and Bendich, A.J. (1994) Extraction of total cellular DNA from plants, algae and fungi. Plant Molecular Biology manual, pp. 183–190. Dordrecht: Springer.

[pbi13492-bib-0048] Rout, K. , Sharma, M. , Gupta, V. , Mukhopadhyay, A. , Sodhi, Y.S. , Pental, D. *et al*. (2015) Deciphering allelic variations for seed glucosinolate traits in oilseed mustard (*Brassica juncea*) using two bi‐parental mapping populations. Theor. Appl. Genet. 128, 657–666. 25628164 10.1007/s00122-015-2461-9

[pbi13492-bib-0049] Rout, K. , Yadav, B.G. , Yadava, S.K. , Mukhopadhyay, A. , Gupta, V. , Pental, D. *et al*. (2018) QTL landscape for oil content in *Brassica juncea*: analysis in multiple bi‐parental populations in high and "0" erucic background. Front. Plant Sci. 9, 1448. 30386353 10.3389/fpls.2018.01448 PMC6198181

[pbi13492-bib-0050] Schranz, M.E. , Lysak, M.A. and Mitchell‐Olds, T. (2006) The ABC's of comparative genomics in the Brassicaceae: building blocks of crucifer genomes. Trends Plant Sci. 11, 535–542. 17029932 10.1016/j.tplants.2006.09.002

[pbi13492-bib-0051] Sharma, R. , Mishra, M. , Gupta, B. , Parsania, C. , Singla‐Pareek, S.L. and Pareek, A. (2015) De novo assembly and characterization of stress transcriptome in a salinity‐tolerant variety CS52 of *Brassica juncea* . PLoS One, 10, e0126783. 25970274 10.1371/journal.pone.0126783 PMC4429966

[pbi13492-bib-0052] Sharma, S. , Padmaja, K.L. , Gupta, V. , Paritosh, K. , Pradhan, A.K. and Pental, D. (2014) Two plastid DNA lineages–Rapa/Oleracea and Nigra–within the tribe Brassiceae can be best explained by reciprocal crosses at hexaploidy: evidence from divergence times of the plastid genomes and R‐block genes of the A and B genomes of Brassica juncea. PLoS One, 9, e93260. 24691069 10.1371/journal.pone.0093260 PMC3972200

[pbi13492-bib-0053] Srivastava, A. , Gupta, V. , Pental, D. and Pradhan, A.K. (2001) AFLP‐based genetic diversity assessment amongst agronomically important natural and some newly synthesized lines of *Brassica juncea* . Theor Appl Genet. 102, 193–199.

[pbi13492-bib-0054] Stanke, M. and Morgenstern, B. (2005) AUGUSTUS: a web server for gene prediction in eukaryotes that allows user‐defined constraints. Nucleic Acids Res. 33, W465–467. 15980513 10.1093/nar/gki458 PMC1160219

[pbi13492-bib-0055] Suyama, M. , Torrents, D. and Bork, P. (2006) PAL2NAL: robust conversion of protein sequence alignments into the corresponding codon alignments. Nucleic Acids Res. 34, W609–612. 16845082 10.1093/nar/gkl315 PMC1538804

[pbi13492-bib-0056] Talavera, G. and Castresana, J. (2007) Improvement of phylogenies after removing divergent and ambiguously aligned blocks from protein sequence alignments. Syst. Biol. 56, 564–577. 17654362 10.1080/10635150701472164

[pbi13492-bib-0057] Tang, H. , Woodhouse, M.R. , Cheng, F. , Schnable, J.C. , Pedersen, B.S. , Conant, G. *et al*. (2012) Altered patterns of fractionation and exon deletions in *Brassica rapa* support a two‐step model of paleohexaploidy. Genetics, 190, 1563–1574. 22308264 10.1534/genetics.111.137349 PMC3316664

[pbi13492-bib-0058] Taylor, J. and Butler, D. (2017) R Package ASMap: Efficient genetic linkage map construction and diagnosis. J. Statis. Soft. 79, 1–29.

[pbi13492-bib-0060] Van Dijk, E.L. , Jaszczyszyn, Y. , Naquin, D. and Thermes, C. (2018) The Third Revolution in Sequencing Technology. Trends Genet. 34, 666–681. 29941292 10.1016/j.tig.2018.05.008

[pbi13492-bib-0061] Walker, B.J. , Abeel, T. , Shea, T. , Priest, M. , Abouelliel, A. , Sakthikumar, S. *et al*. (2014) Pilon: an integrated tool for comprehensive microbial variant detection and genome assembly improvement. PLoS One, 9, e112963. 25409509 10.1371/journal.pone.0112963 PMC4237348

[pbi13492-bib-0062] Wang, Y. , Tang, H. , Debarry, J.D. , Tan, X. , Li, J. , Wang, X. *et al*. (2012) MCScanX: a toolkit for detection and evolutionary analysis of gene synteny and collinearity. Nucleic Acids Res. 40, e49. 22217600 10.1093/nar/gkr1293 PMC3326336

[pbi13492-bib-0063] Wang, X. , Wang, H. , Wang, J. , Sun, R. , Wu, J. *et al*. (2011) The genome of the mesopolyploid crop species *Brassica rapa* . Nat. Genet. 43, 1035–1039. 21873998 10.1038/ng.919

[pbi13492-bib-0064] Warwick, S.I. and Black, L.D. (1991) Molecular systematics of Brassica and allied genera (subtribe Brassicinae, Brassiceae)—chloroplast genome and cytodeme congruence. Theor. Appl. Genet. 82, 81–92. 24212864 10.1007/BF00231281

[pbi13492-bib-0065] Wicker, T. , Sabot, F. , Hua‐Van, A. , Bennetzen, J.L. , Capy, P. , Chalhoub, B. *et al*. (2007) A unified classification system for eukaryotic transposable elements. Nat. Rev. Genet. 8, 973–982. 17984973 10.1038/nrg2165

[pbi13492-bib-0066] Wu, Y. , Bhat, P.R. , Close, T.J. and Lonardi, S. (2008) Efficient and accurate construction of genetic linkage maps from the minimum spanning tree of a graph. PLoS Genet. 4, e1000212. 18846212 10.1371/journal.pgen.1000212 PMC2556103

[pbi13492-bib-0067] Xu, Z. and Wang, H. (2007) LTR_FINDER: an efficient tool for the prediction of full‐length LTR retrotransposons. Nucleic Acids Res. 35, W265–268. 17485477 10.1093/nar/gkm286 PMC1933203

[pbi13492-bib-0068] Yadava, S.K. , Arumugam, N. , Mukhopadhyay, A. , Sodhi, Y.S. , Gupta, V. , Pental, D. and Pradhan, A.K. (2012) QTL mapping of yield‐associated traits in *Brassica juncea*: meta‐analysis and epistatic interactions using two different crosses between east European and Indian gene pool lines. Theor. Appl. Genet. 125, 1553–1564. 22821338 10.1007/s00122-012-1934-3

[pbi13492-bib-0069] Yang, Z. (2007) PAML 4: phylogenetic analysis by maximum likelihood. Mol. Biol. Evol. 24, 1586–1591. 17483113 10.1093/molbev/msm088

[pbi13492-bib-0070] Yang, J. , Ji, C. , Liu, D. , Wang, X. and Zhang, M. (2018) Reply to: 'Organization of the genome sequence of the polyploid crop species *Brassica juncea'* . Nat. Genet. 50, 1497–1498. 30250129 10.1038/s41588-018-0240-7

[pbi13492-bib-0071] Yang, J. , Liu, D. , Wang, X. , Ji, C. , Cheng, F. , Liu, B. *et al*. (2016) The genome sequence of allopolyploid *Brassica juncea* and analysis of differential homoeolog gene expression influencing selection. Nat. Genet. 48, 1225–1232. 27595476 10.1038/ng.3657

[pbi13492-bib-0072] Zhang, L. , Cai, X. , Wu, J. , Liu, M. , Grob, S. , Cheng, F. *et al*. (2018) Improved *Brassica rapa* reference genome by single‐molecule sequencing and chromosome conformation capture technologies. Hortic. Res. 5, 50. 30131865 10.1038/s41438-018-0071-9 PMC6092429

[pbi13492-bib-0073] Zimin, A.V. , Marcais, G. , Puiu, D. , Roberts, M. , Salzberg, S.L. and Yorke, J.A. (2013) The MaSuRCA genome assembler. Bioinformatics, 29, 2669–2677. 23990416 10.1093/bioinformatics/btt476 PMC3799473

