# Supplementary material for: A chromosome‐scale assembly of allotetraploid Brassica juncea (AABB) elucidates comparative architecture of the A and B genomes
Source: Plant Biotechnol J. 2020 Dec 30;19(3):602–14. doi: 10.1111/pbi.13492 (PMC7955877; doi:10.1111/pbi.13492)
Supplement: Supplementary file 1 — Figure S1. Genetic map of Brassica juncea Varuna x Heera (VH) F1DH population. Figure S2. Genetic map of Brassica juncea Tumida x Varuna (TuV) F1DH population. Figure S3. Genetic map of Brassica nigra Sangam x 2782 F1DH population. Figure S4. Relationship between the GBS markers on the genetic map of Brassica nigra Sangam x 2782 F1DH population and physical position of the respective marker tags on the B. nigra genomic sequences. Figure S5. Workflow in BioNano optical mapping based hierarchical scaffolding analysis of Brassica juncea genome. Figure S6. Relationship between the GBS markers on the Brassica juncea VH (Varuna x Heera) F1DH population linkage map, and physical position of the respective marker tags on the assembled Varuna genome. Figure S7. Relationship between the GBS markers on the Brassica juncea Tumida x Varuna F1DH population genetic map and the physical position of the respective GBS markers on the assembled Varuna genome. Figure S8. Distribution of different types of transposable elements on Brassica juncea pseudochromosomes. Figure S9. Position of the predicted A genome centromeric sequences on different pseudochromosomes of the A genome of Brassica juncea. Figure S10. Position of the predicted B genome centromeric sequences on different pseudochromosomes of the B genome of Brassica juncea. Figure S11a. Comparison of the genome assembly of Brassica juncea Varuna with previous assemblies of B. rapa Chiifu for the A genome and B. juncea Tumida for both the A and B genomes. Figure S11b. Comparative gene‐to‐gene based collinearity analysis of the Brassica jucnea Varuna genome with B. rapa Chiifu V3.0 assembly and B. nigra Sangam long‐read assembly. Figure S12. Gene density on Brassica juncea A genome pseudochromosomes. Figure S13. Gene density on Brassica juncea B genome pseudochromosomes. Figure S14. Proportion of orthologous genes retained in the A and B genomes of Brassica juncea. Figure S15. Relationship of the three paleogenomes (LF, MF1, and MF [file PBI-19-602-s004.pdf]

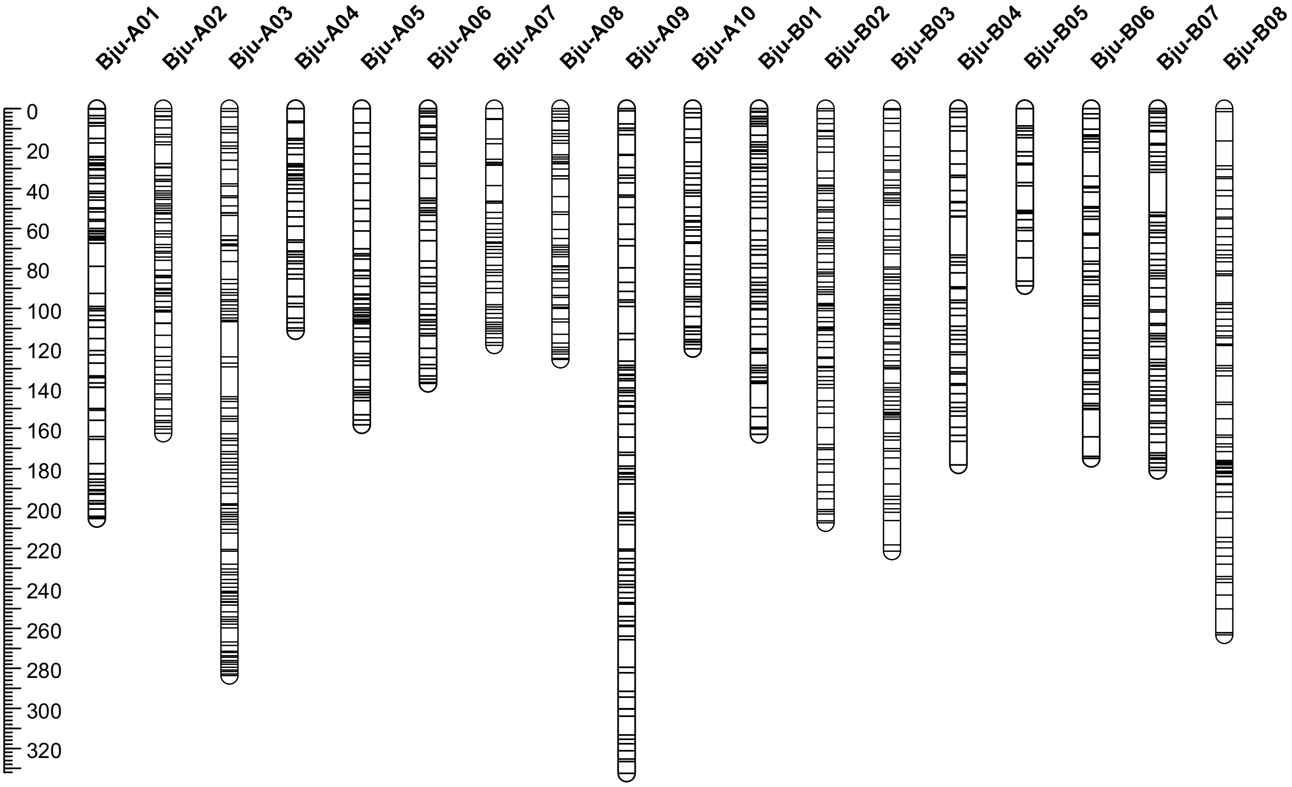

Figure S1. Genetic map of *Brassica juncea* Varuna x Heera (VH) F_1DH population. The LGs have been named as Bju-A01 to Bju-A10, and Bju-B01 to Bju-B08 (Panjabi et al., 2008; Paritosh et al., 2014). The black bands in the LG bar represent the mapped markers. A total of 3,780 markers were mapped on the 18 LGs; these include 833 anchor markers and 2,947 GBS based SNPs derived from *Sph*I-*Mluc*I restriction enzyme combination. The map covered a total genetic length of 3,232.3 cM. Sidebar, cM.

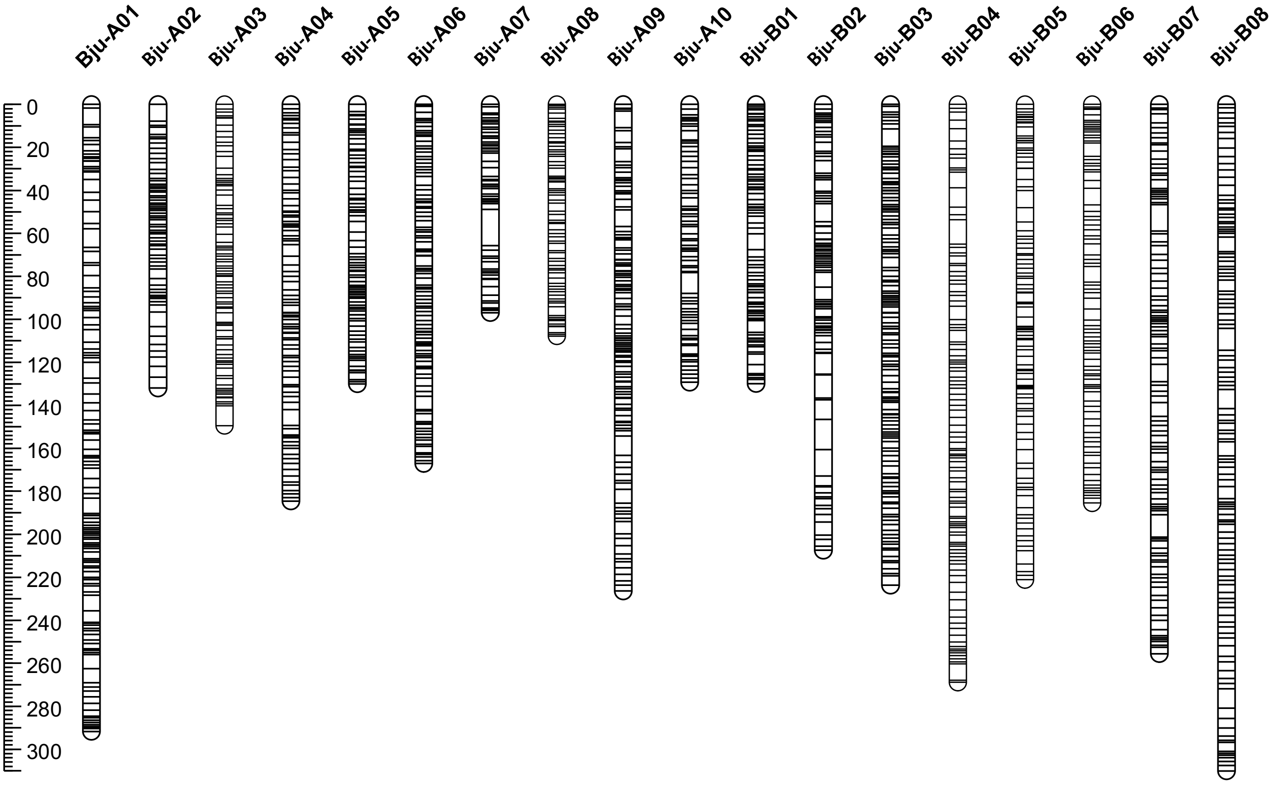

Figure S2. Genetic map of *Brassica juncea* Tumida x Varuna (TuV) F$_1$DH population. The LGs belonging to the A genome were named as Bju-A01 to Bju-A10, and those to the B genome as Bju-B01 to Bju-B08 (Panjabi et al., 2008; Paritosh et al., 2014). The black bands in the LGs represent the mapped markers. A total of 9,041 markers – 524 anchor markers common with the VH population, and 8,517 GBS based SNPs generated using the *Hinf*I-*Hpy*CH4IV enzyme combination were mapped on 18 LGs. The map covered a total genetic length of 3,417.4 cM. Sidebar, cM.

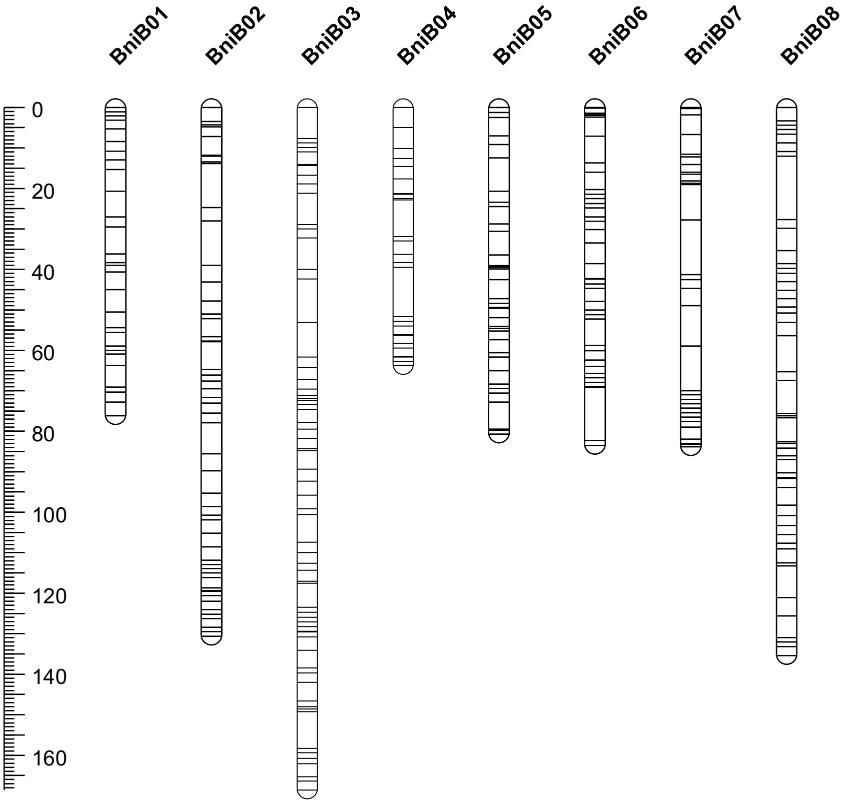

Figure S3. Genetic map of *Brassica nigra* Sangam x 2782 F_1DH population. The LGs have been named as Bni-B01 to Bni-B08 (Panjabi et al., 2008; Paritosh et al., 2014). The black bands in the LGs represent the mapped markers that covered a total genetic length of 822.6 cM. Only a subset of 1,765 uniquely placed markers have been shown on the eight LGs. The map incorporates 197 anchor markers common with the VH population, and 1,568 GBS-based SNPs generated using the *Sph*I-*Mluc*I enzyme combination. Sidebar, cM.

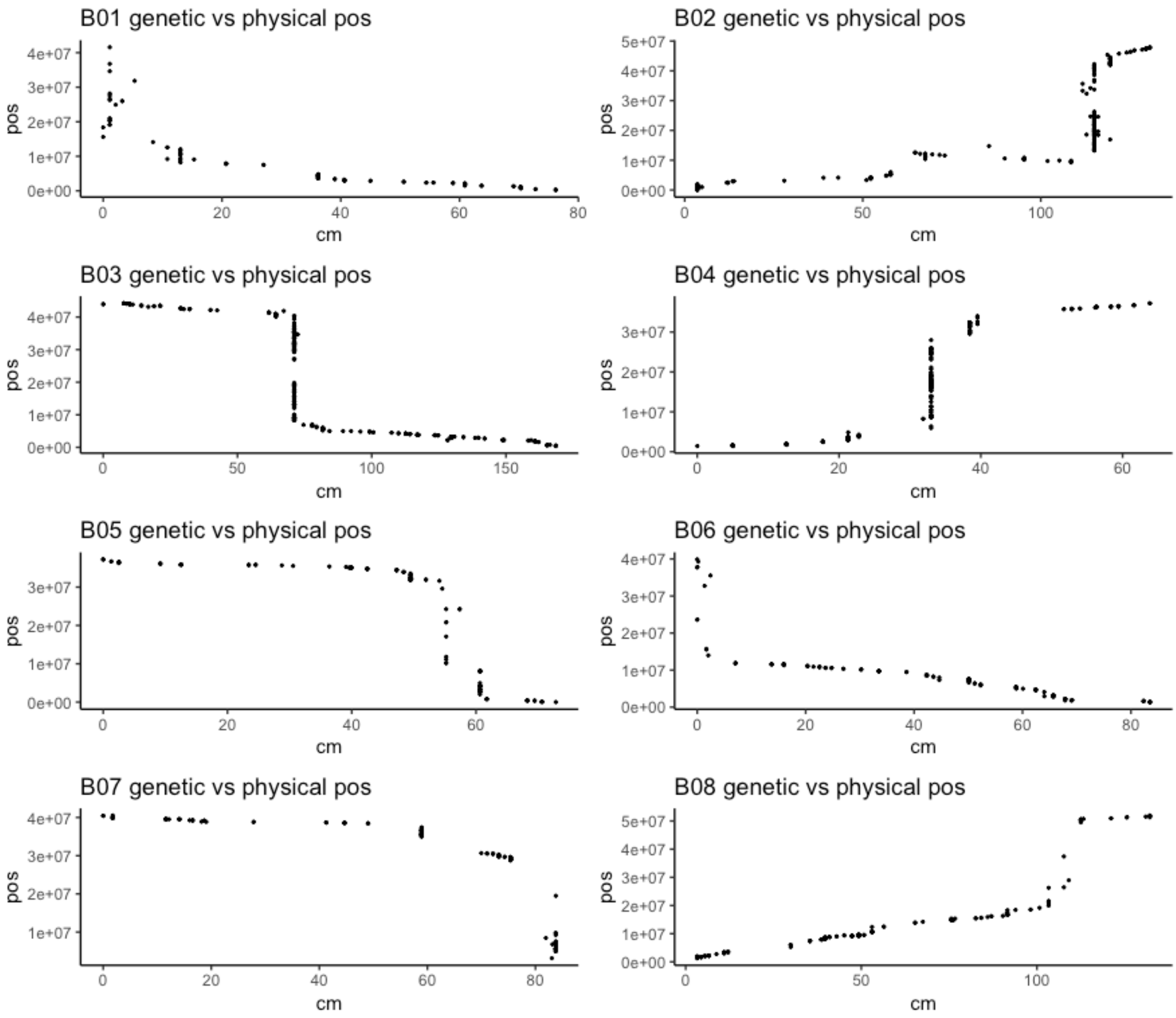

Figure S4. Relationship between the GBS markers on the genetic map of *Brassica nigra* Sangam x 2782 F_1DH population and physical position of the respective marker tags on the *B. nigra* genomic sequences. Genetic positions of the markers have been shown on the x-axis and physical positions on the y-axis. A linear relationship was found between the physical and genetic distances of the markers present on the LGs except at the centromeric regions where the rate of recombination would be much lower than in the other chromosomal regions.

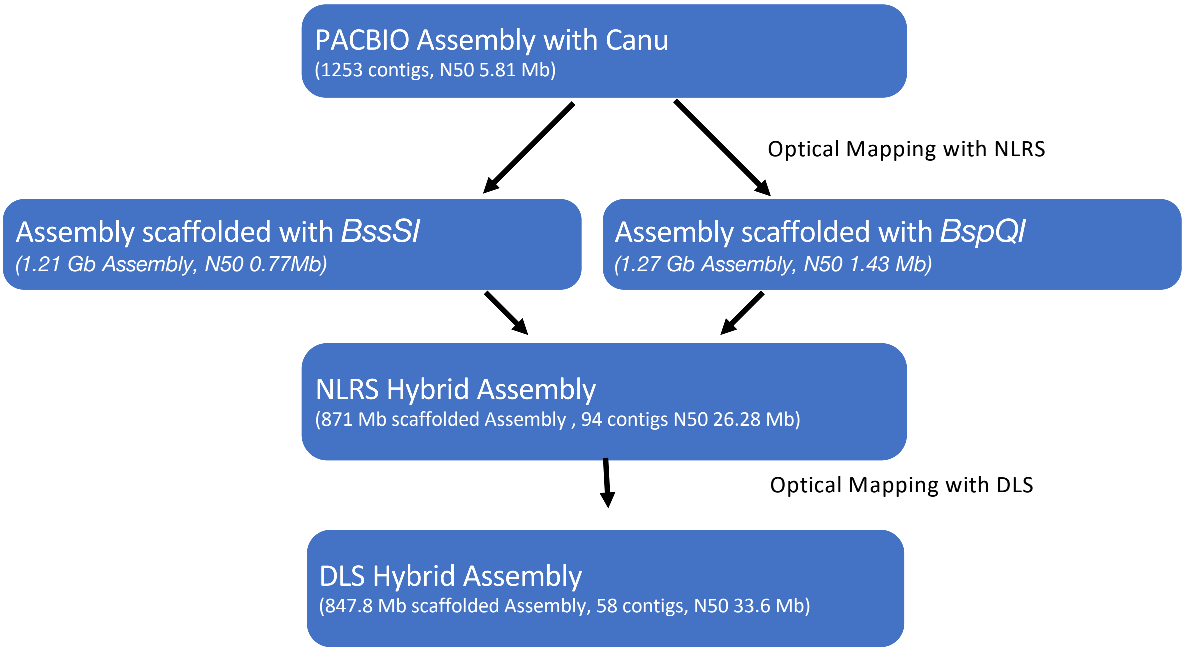

Figure S5. Workflow in BioNano optical mapping based hierarchical scaffolding analysis of *Brassica juncea* genome. Two NLRS based labeling reactions were carried out, c-maps were generated, and the assembled PacBio contigs were aligned to these. A hybrid assembly of the two c-maps and corrected PacBio contigs yielded 94 scaffolds, covering ~871 Mb of the genome. The assembled scaffolds were aligned again with the DLS maps to generate an 847.8 Mb assembly consisting of 58 scaffolds.

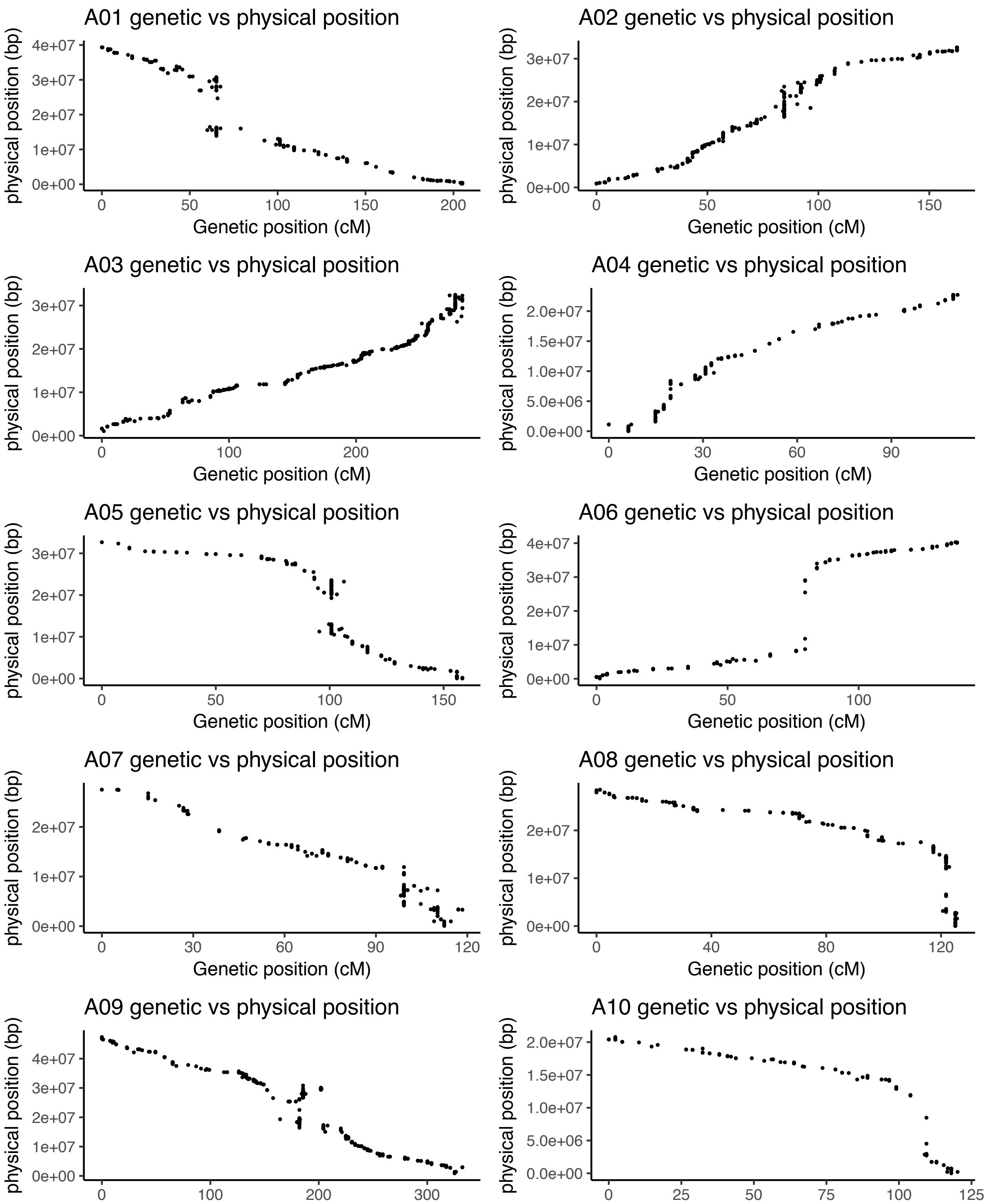

Figure S6. Relationship between the GBS markers on the *Brassica juncea* VH (Varuna x Heera) F1DH population linkage map, and physical position of the respective marker tags on the assembled Varuna genome. Genetic positions of the markers have been shown on the x-axis, and physical positions are shown on the y-axis. A linear relationship was found between the physical and genetic distances of the markers present on an LG except at the centromeric region where the rate of recombination would be much lower than the other chromosomal regions.

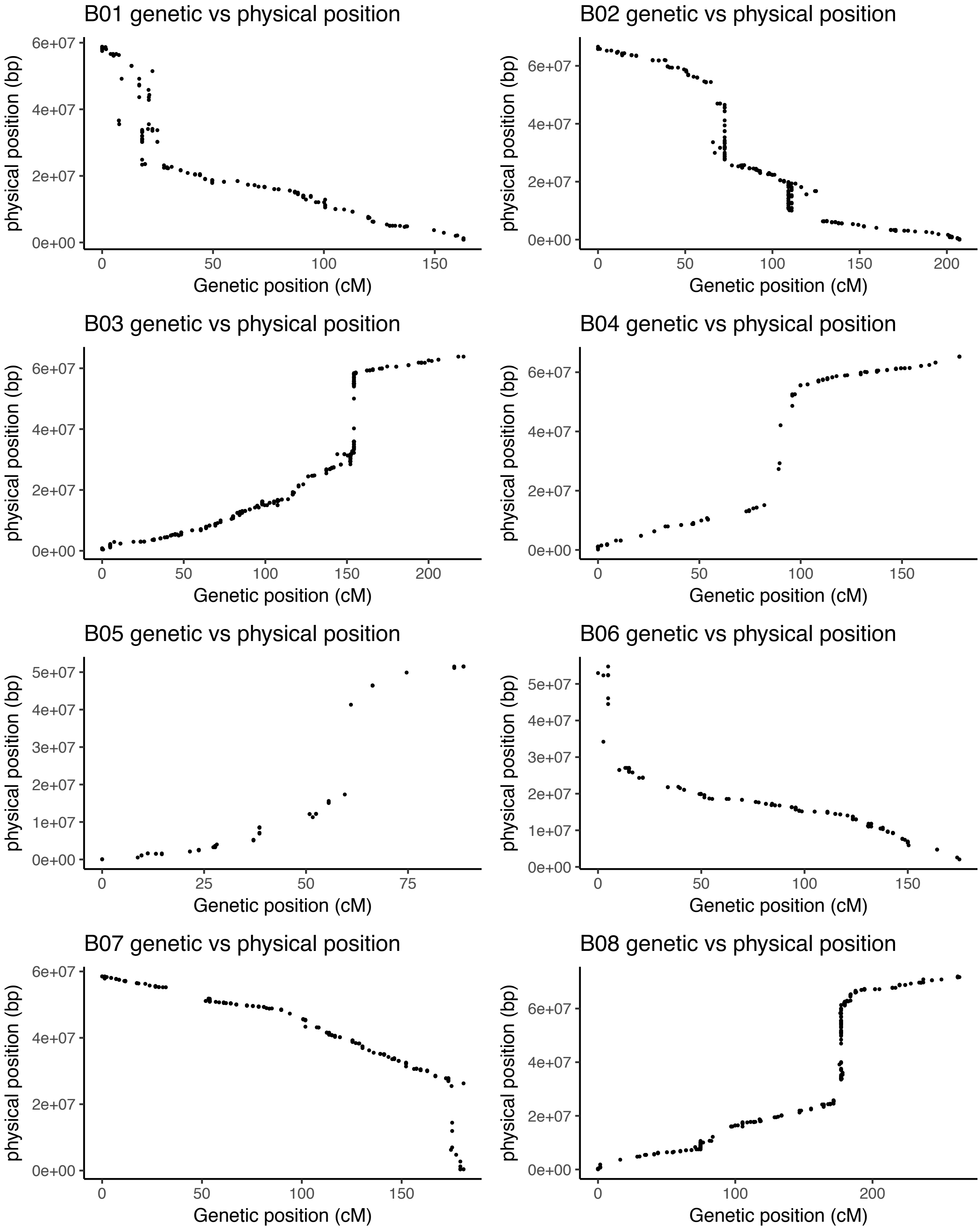

Figure S6. contd..

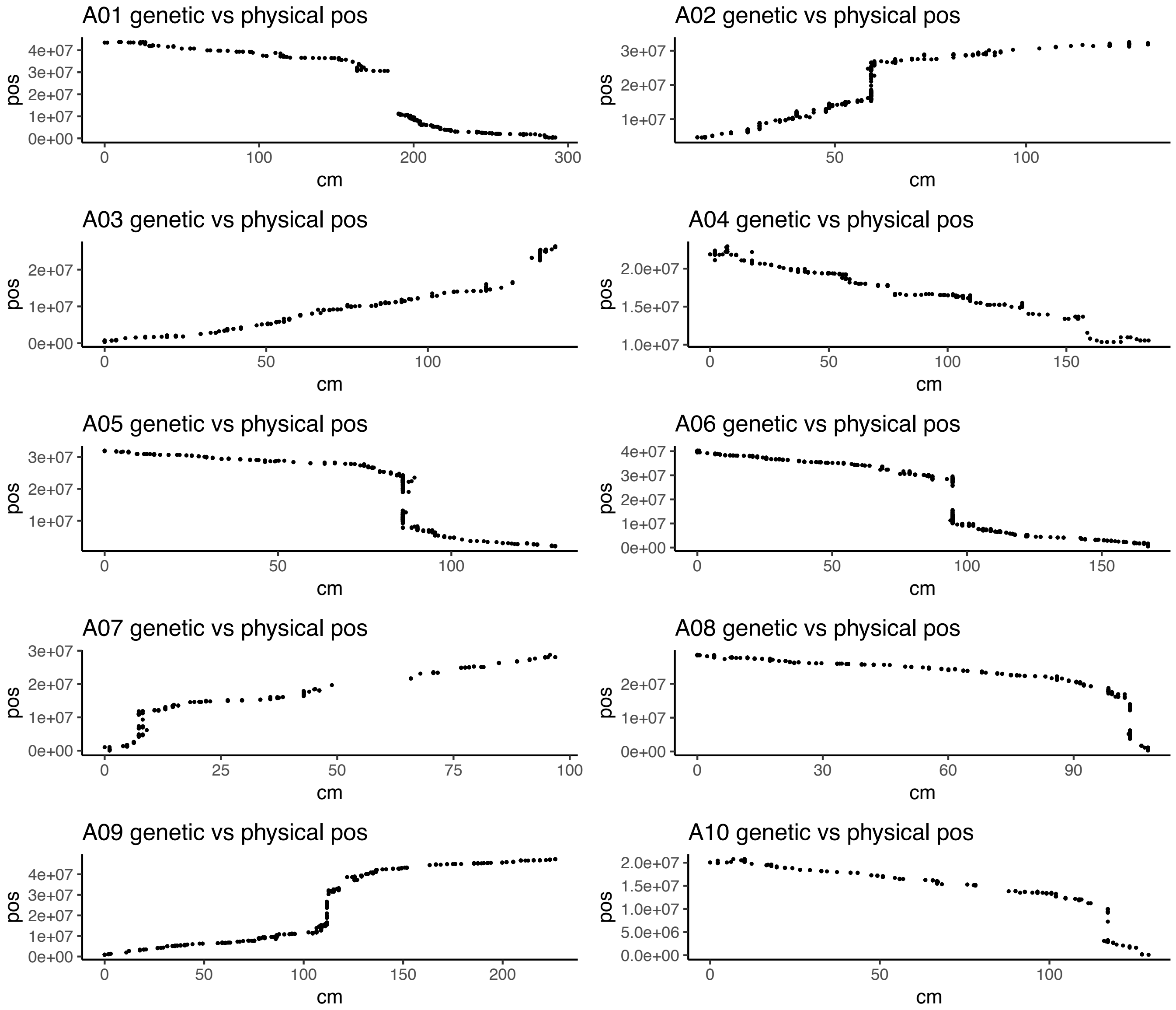

Figure S7. Relationship between the GBS markers on the *Brassica juncea* Tumida x Varuna F_1DH population genetic map and the physical position of the respective GBS markers on the assembled Varuna genome. Genetic positions of the markers in centimorgan (cM) have been shown on the x-axis, and the physical positions are shown on the y-axis. A linear relationship was found between the physical and genetic positions of the makers except at the centromeric regions. A region of the B07 pseudochromosome could not be covered with the linkage map analysis as no polymorphic marker could be detected between Tumida and Varuna parental lines in the first 30Mb region of the LG B07.

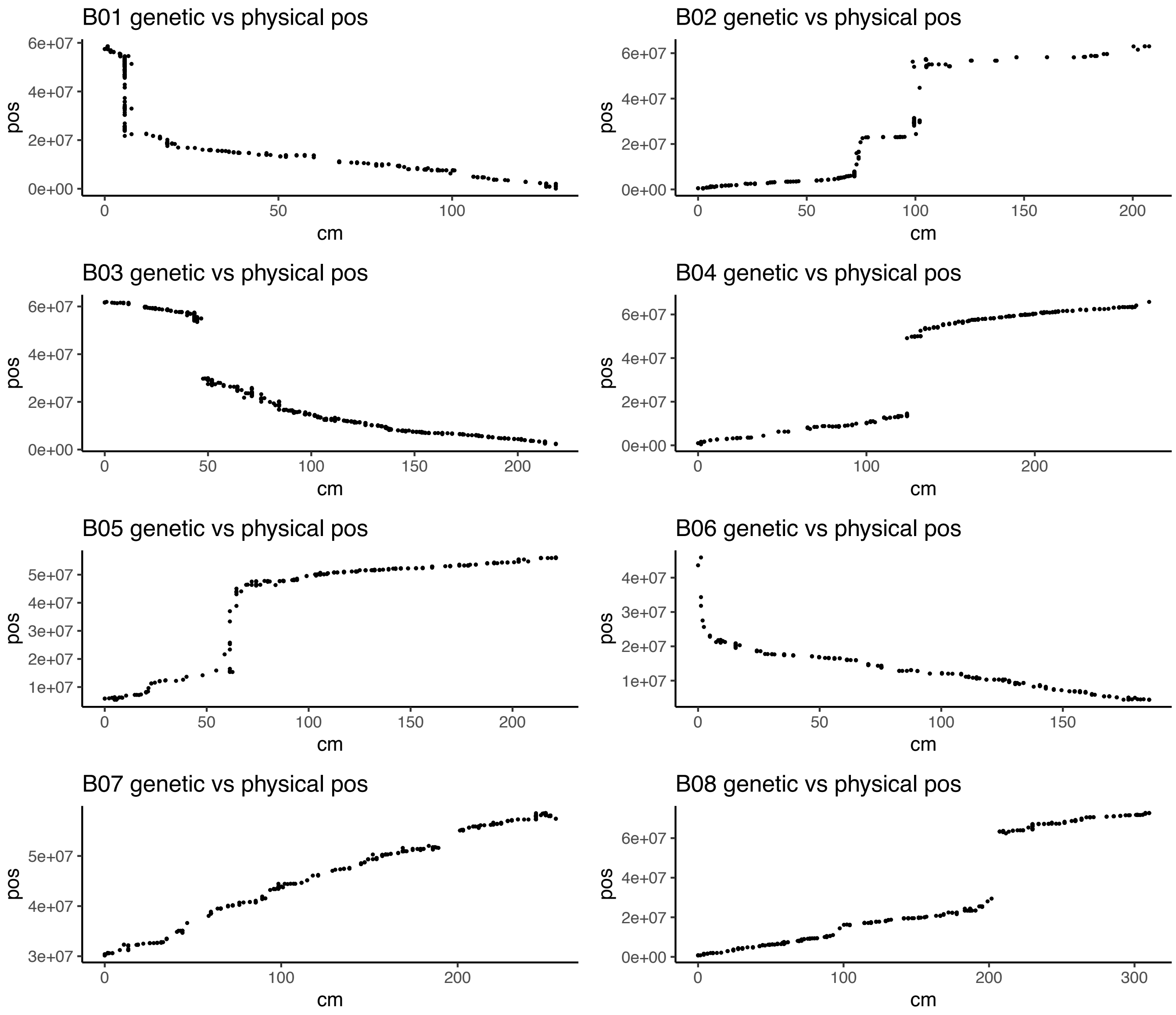

Figure S7. contd..

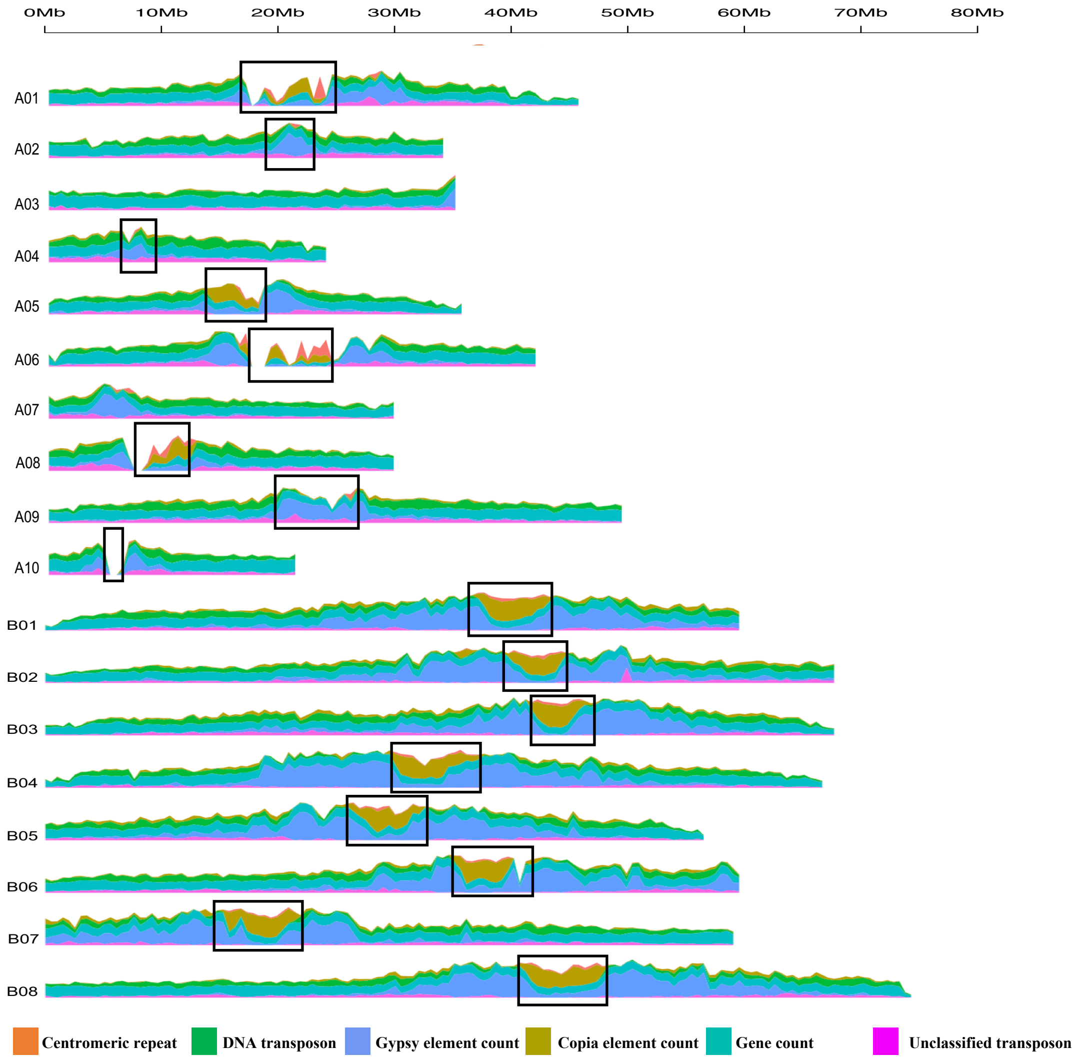

Figure S8. Distribution of different types of transposable elements on *Brassica juncea* pseudochromosomes. Centromeric regions are boxed and show much higher content of TEs in the B genome.

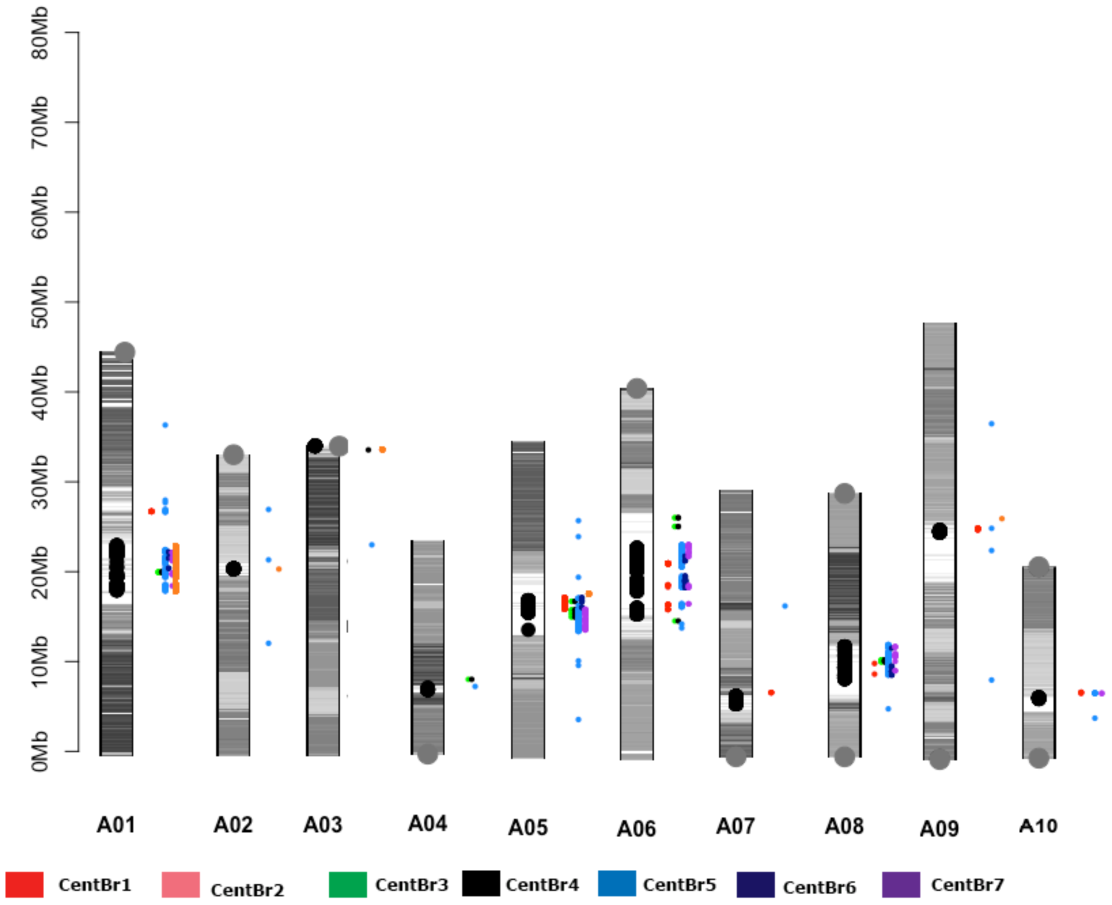

Figure S9. Position of the predicted A genome centromeric sequences on different pseudochromosomes of the A genome of *Brassica juncea*. The number of dots shows the extent of repeats of a sequence on the pseudochromosomes. Different centromere-specific sequences have been assigned different colors.

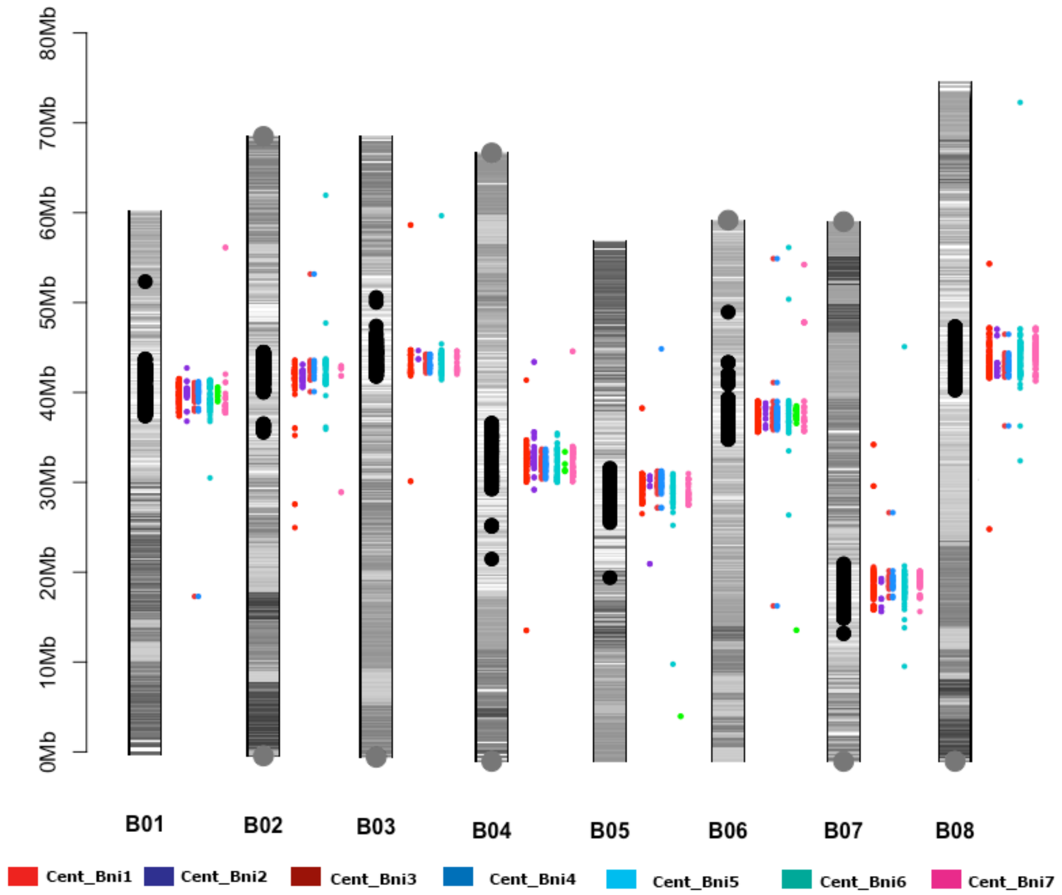

Figure S10. Position of the predicted B genome centromeric sequences on different pseudochromosomes of the B genome of *Brassica juncea*. Different centromere-specific sequences have been assigned different colors. The number of dots shows the extent of repetition on the pseudochromosomes.

Comparison of A01- Chiifu and Tumida assemblies vs Varuna assembly

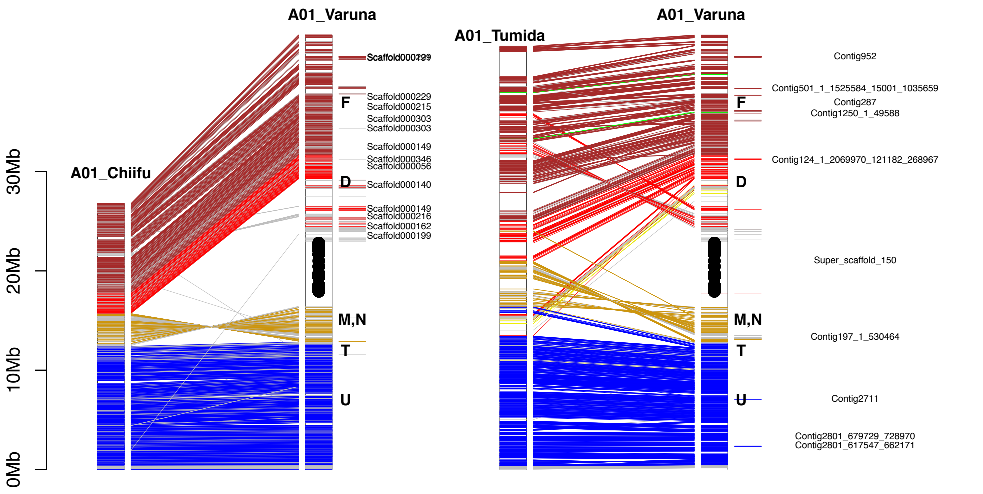

Comparison of A02- Chiifu and Tumida assemblies vs Varuna assembly

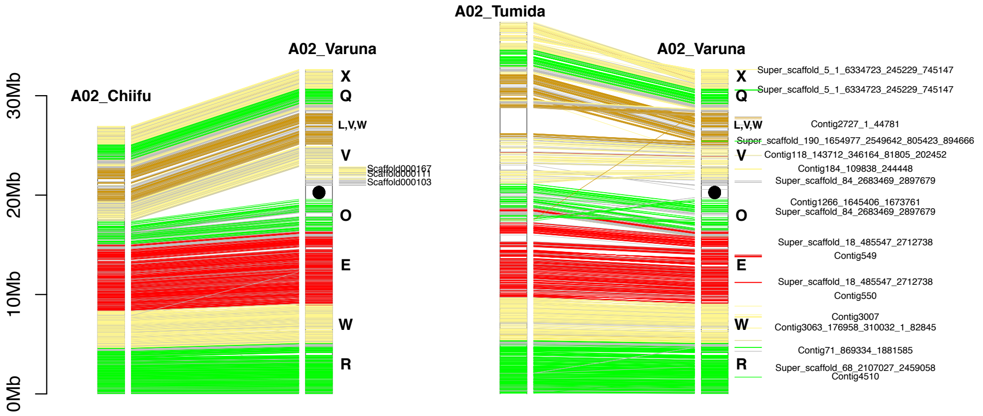

Figure S11a. Comparison of the genome assembly of *B. juncea* Varuna with previous assemblies of *B. rapa* Chiifu (Wang et al., 2011) for the A genome and *B. juncea* variety Tumida (Yang et al., 2016) for both the A and B genomes. Comparisons were made based on the gene order on each pseudochromosome in the three assemblies, each gene is represented by a line. Scaffolds and contigs of the earlier assemblies that were unassigned to any pseudochromosomes could be assigned in the Varuna assembly (shown on the right of the Varuna pseudochromosomes; only some have been shown due to inadequate space). The A genome pseudochromosomes of Varuna matched the Chiifu assembly more than the Tumida assembly.

Comparison of A03- Chiifu and Tumida assemblies vs Varuna assembly

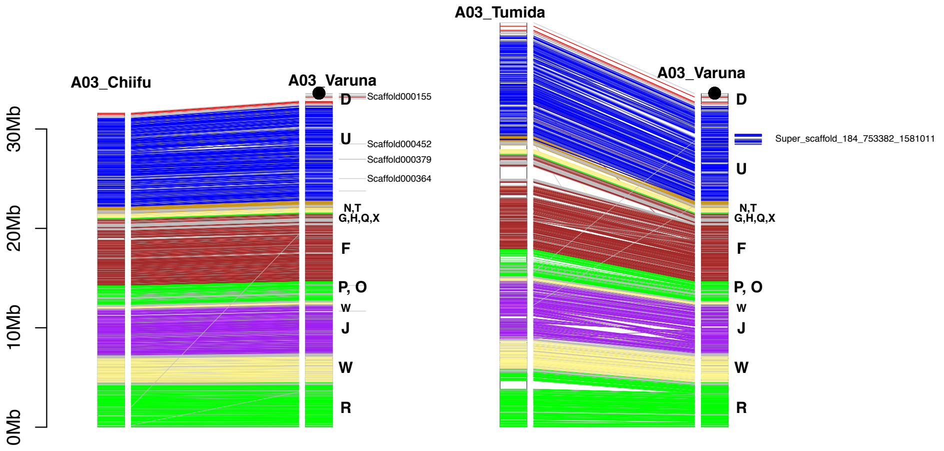

Comparison of A04- Chiifu and Tumida assemblies vs Varuna assembly

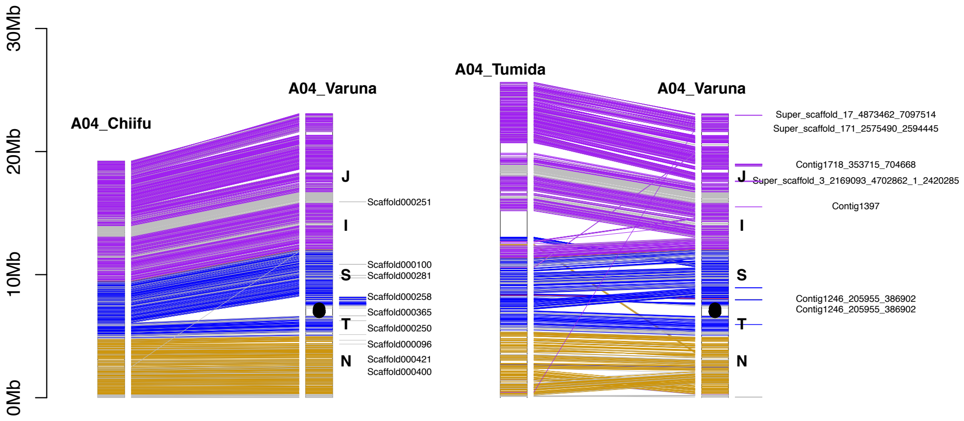

Figure S11a. contd..

Comparison of A05- Chiifu and Tumida assemblies vs Varuna assembly

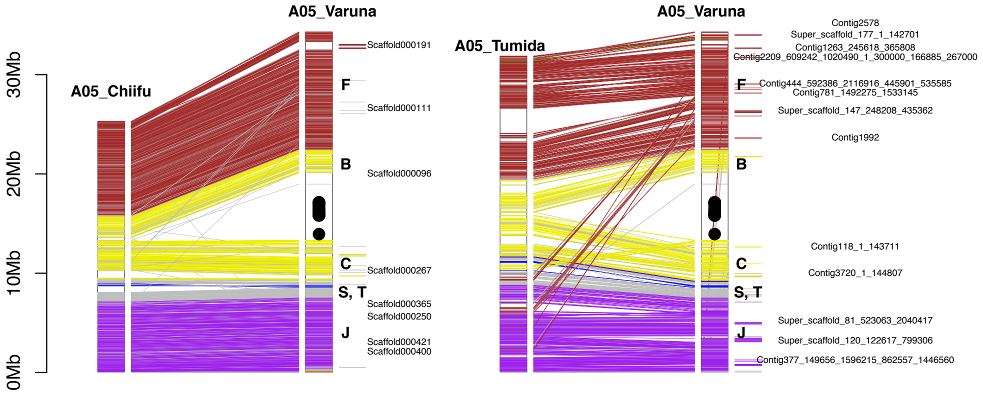

Comparison of A06- Chiifu and Tumida assemblies vs Varuna assembly

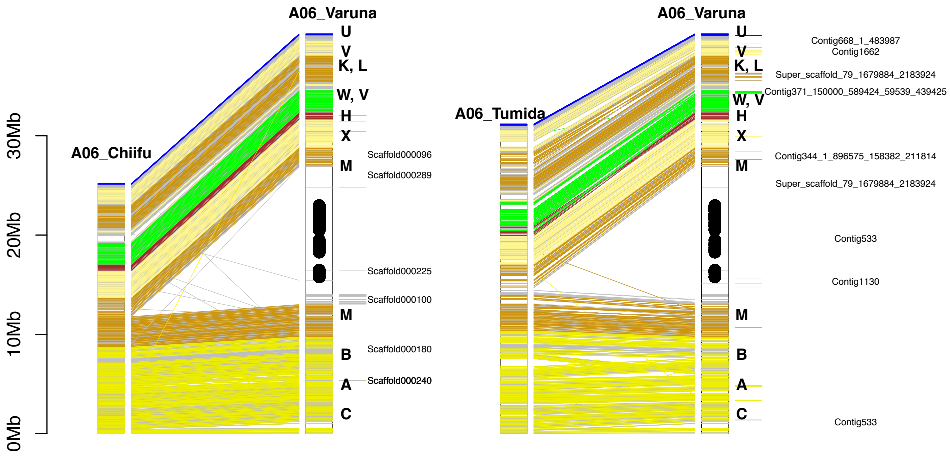

Figure S11a. contd..

Comparison of A07- Chiifu and Tumida assemblies vs Varuna assembly

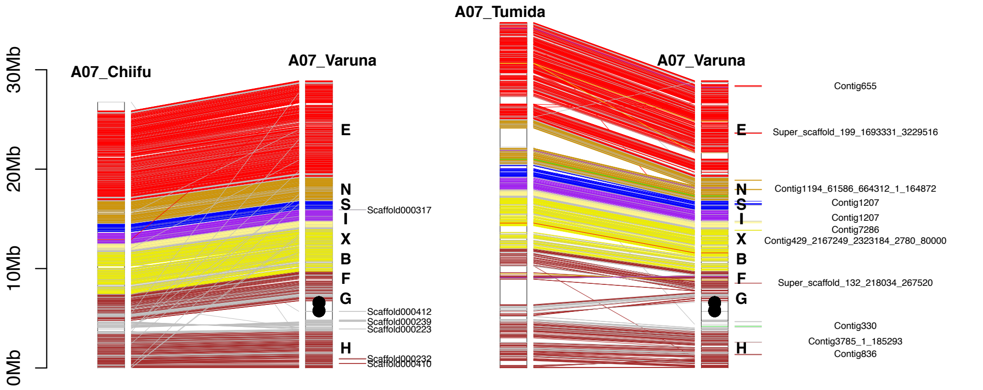

Comparison of A08- Chiifu and Tumida assemblies vs Varuna assembly

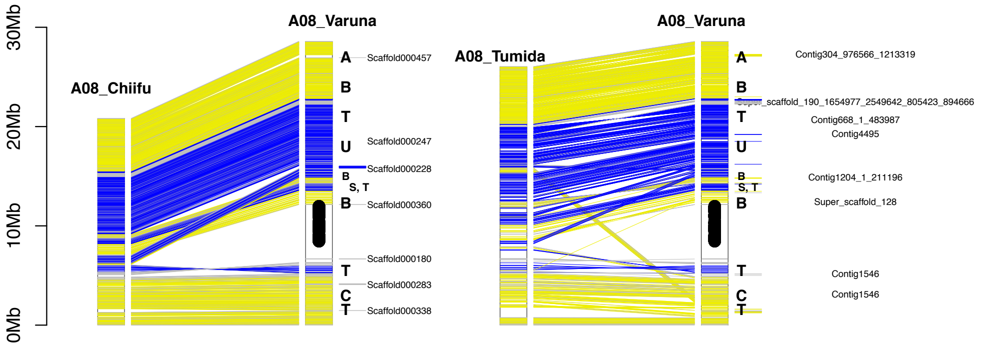

Figure S11a. contd..

Comparison of A09- Chiifu and Tumida assemblies vs Varuna assembly

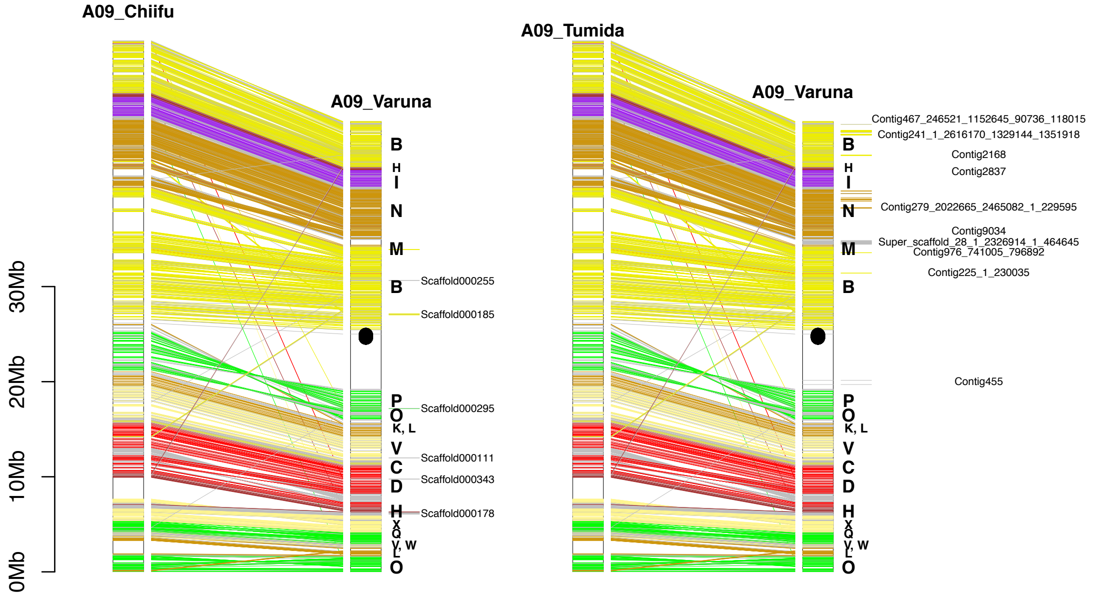

Comparison of A10- Chiifu and Tumida assemblies vs Varuna assembly

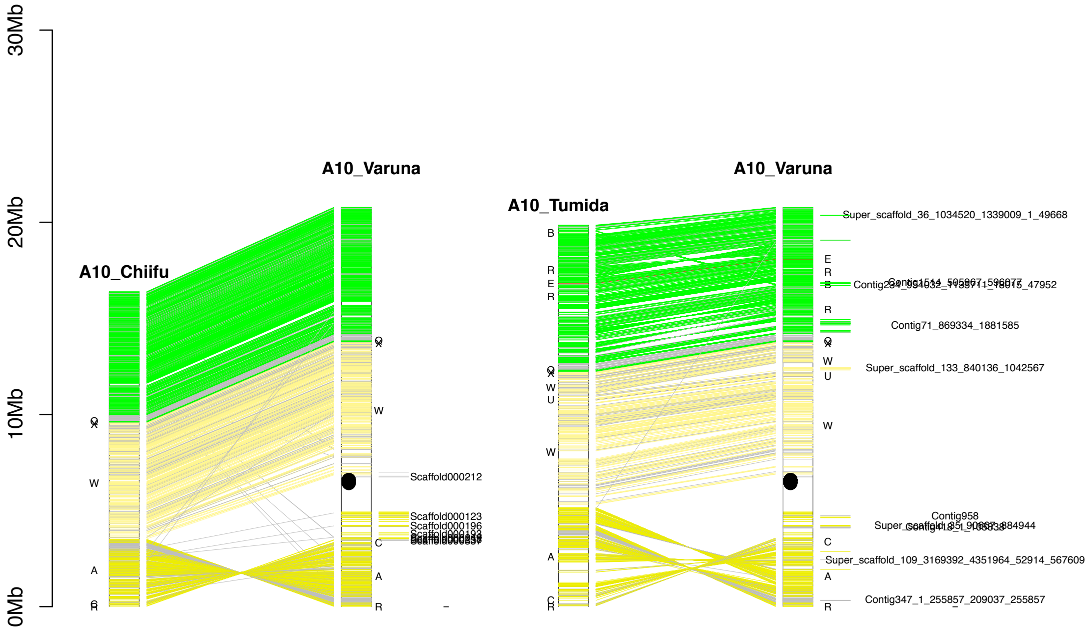

Figure S11a. contd..

Comparison of B01 - B04 pseudochromosomes- Tumida vs Varuna assembly

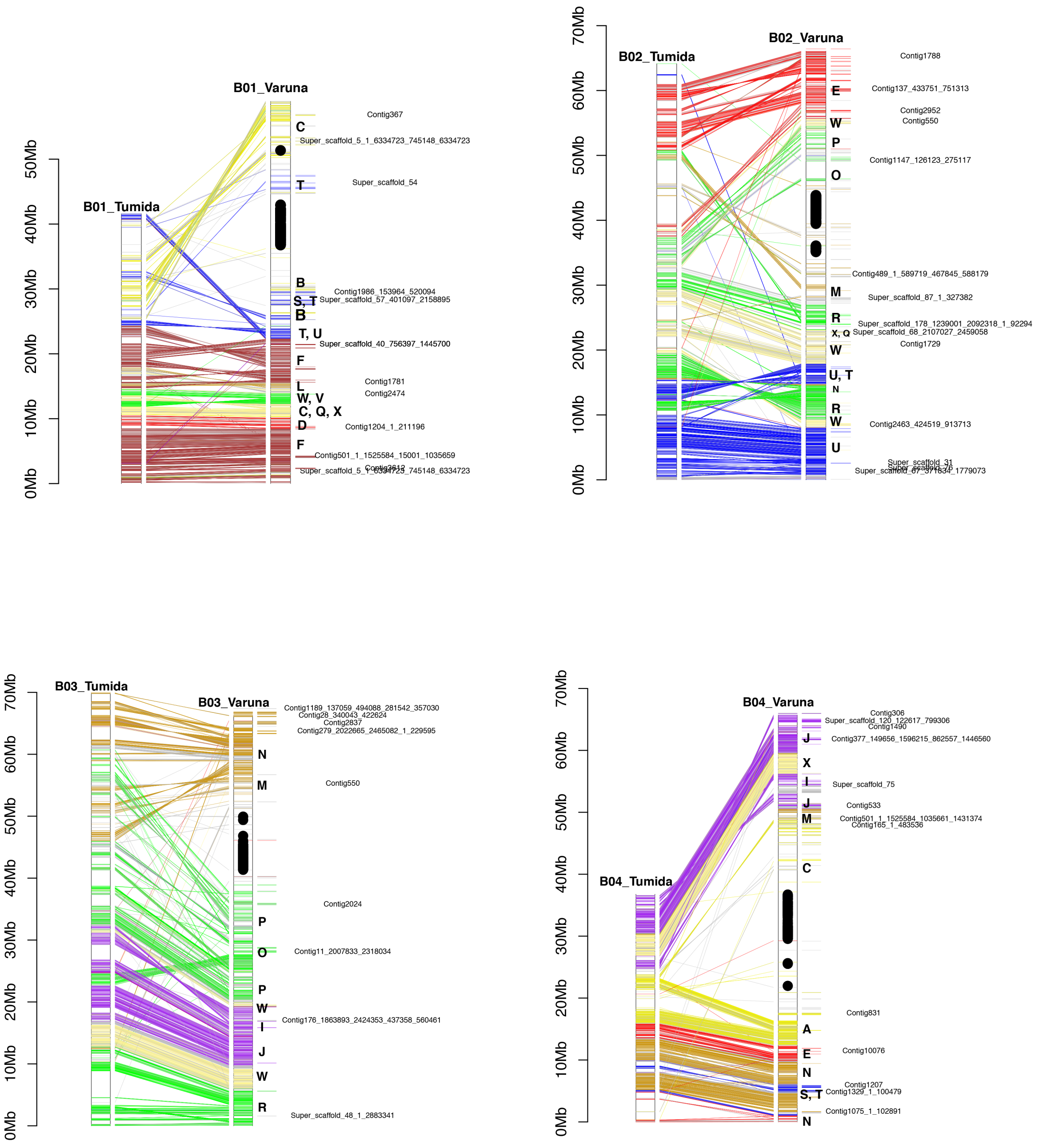

Figure S11a. contd..

Comparison of B05 - B08 pseudochromosomes- Tumida vs Varuna assembly

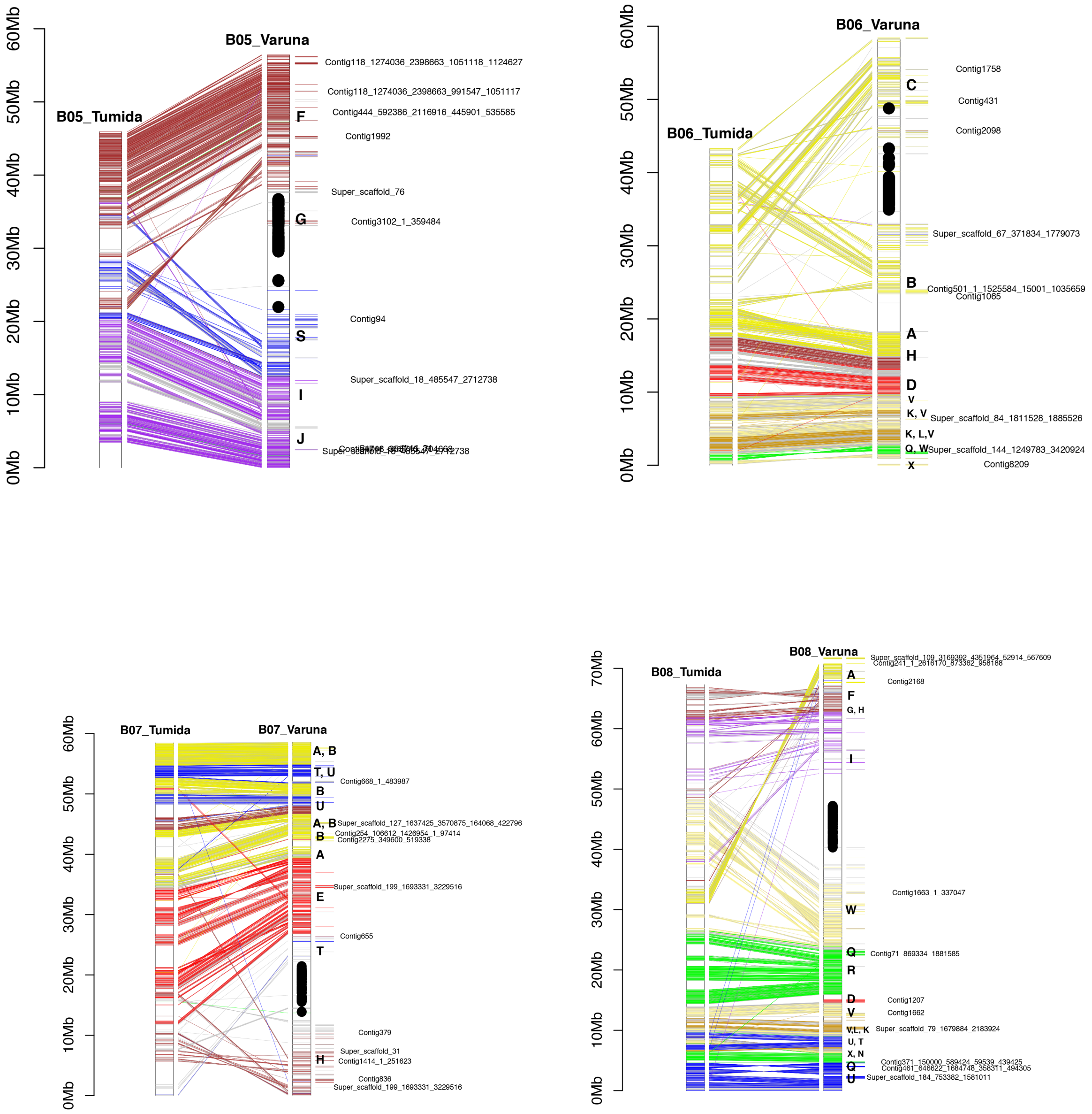

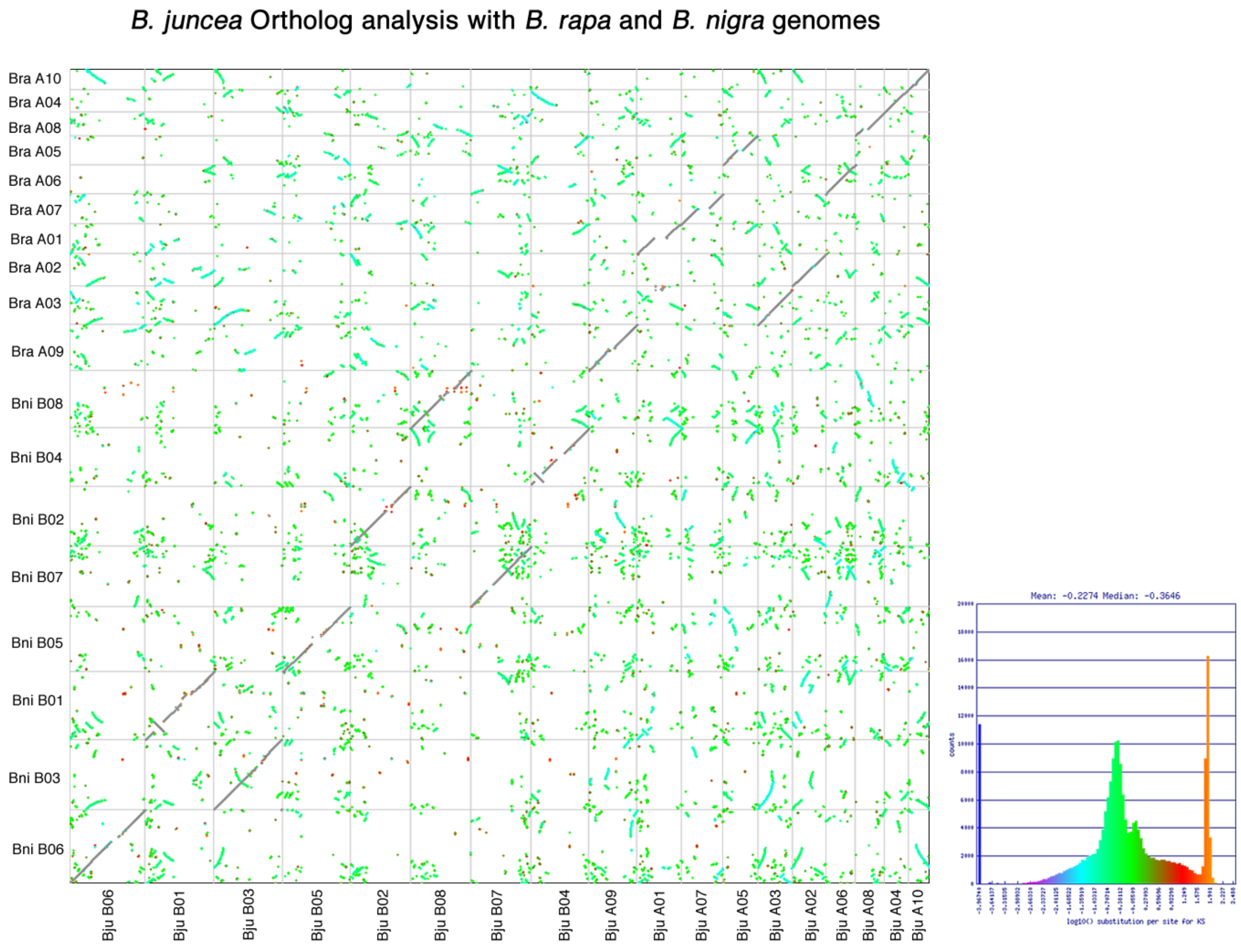

Figure S11b. Comparative gene-to-gene based collinearity analysis of the assembled *B. jucnea* variety Varuna genome with *B. rapa* Chiifu V3.0 assembly (Zhang et al., 2018) and *B. nigra* Sangam long read assembly (Paritosh et al., 2020). Very high collinearity is present between the BjuA and BraA, and BjuB and BniB genomes. Analysis was carried out using Synmap program at CoGe webserver. Each blue/grey dot represents an orthologous gene identified using least Ks value based divergence. Centromeric regions, devoid of any functional genes, are represented by gaps. No exchanges (≥5 gene region) were observed between the homoeologous regions of BjuA and BjuB genomes.

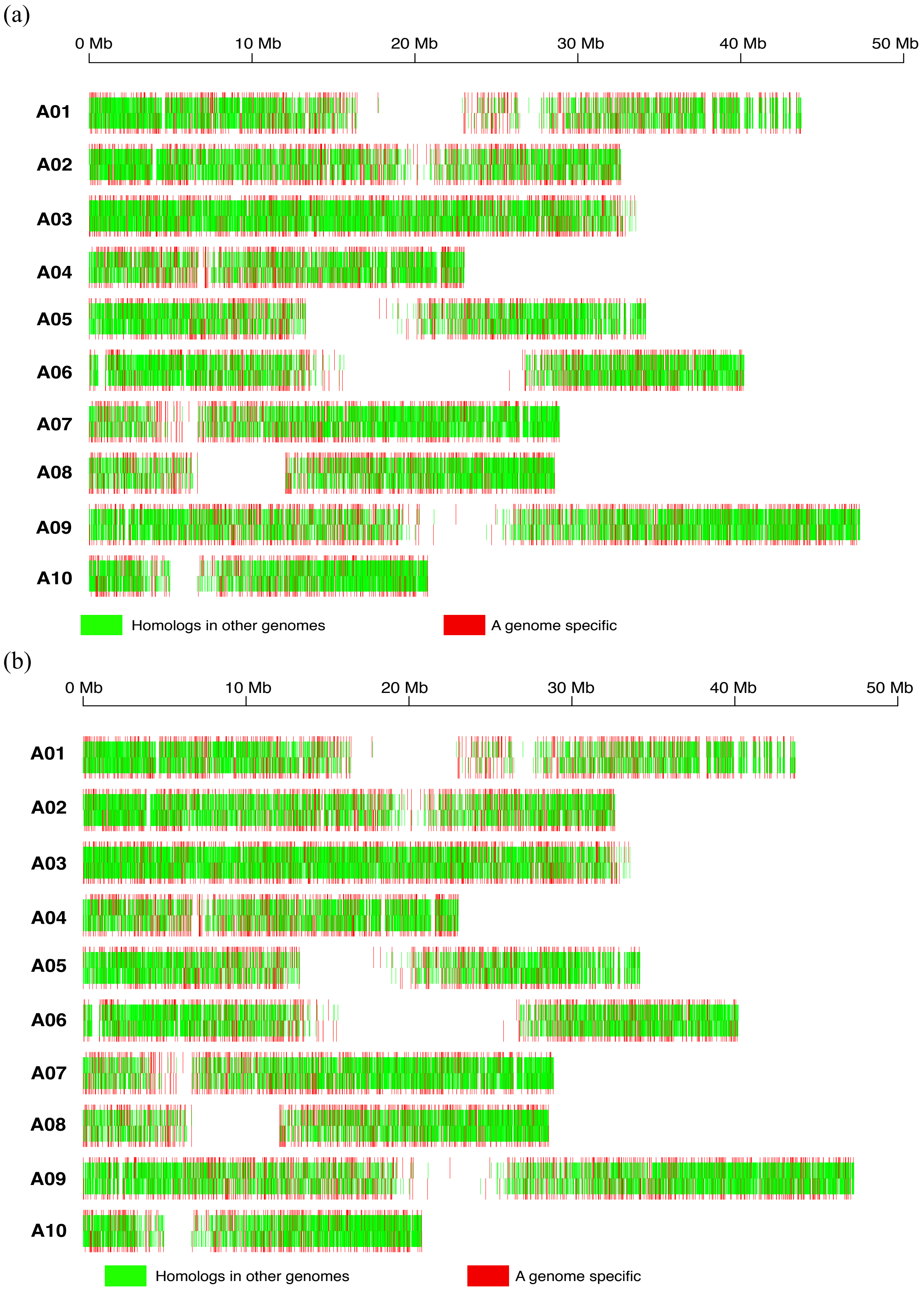

Figure S12. Gene density on *B. juncea* A genome pseudochromosomes. Green bars show genes that have orthologs in the B genome of *B. juncea* and *A. thaliana*. Red lines represent the A genome-specific genes – (a) total predicted genes (total 8,934) on A01-A10 pseudochromosomes (b) genes that were found to express in the transcriptome data (total 4,129).

(a)

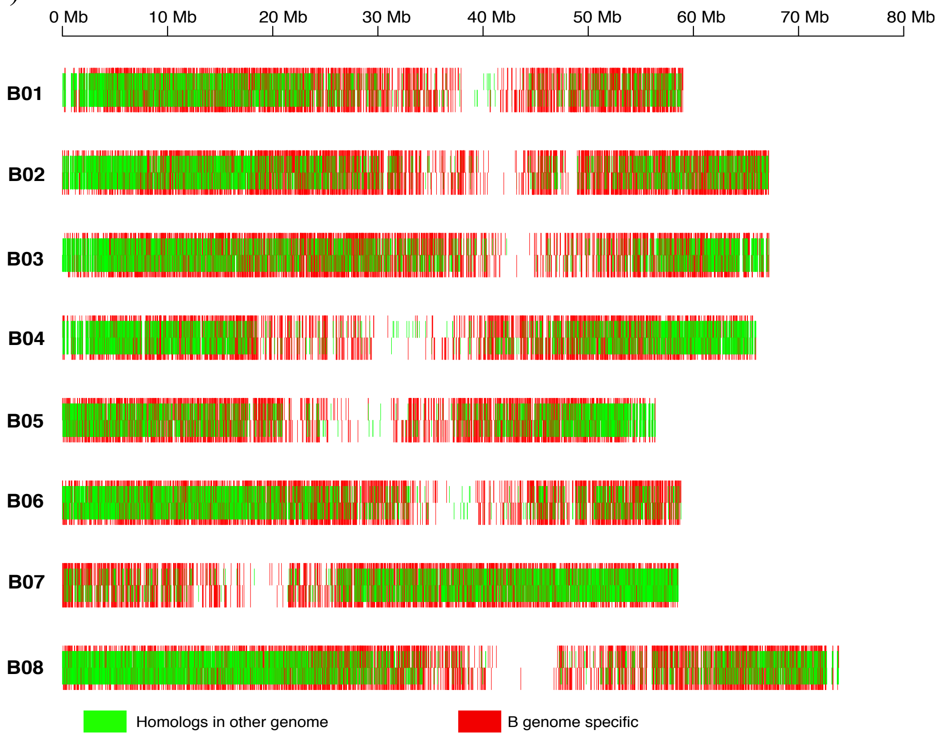

(b)

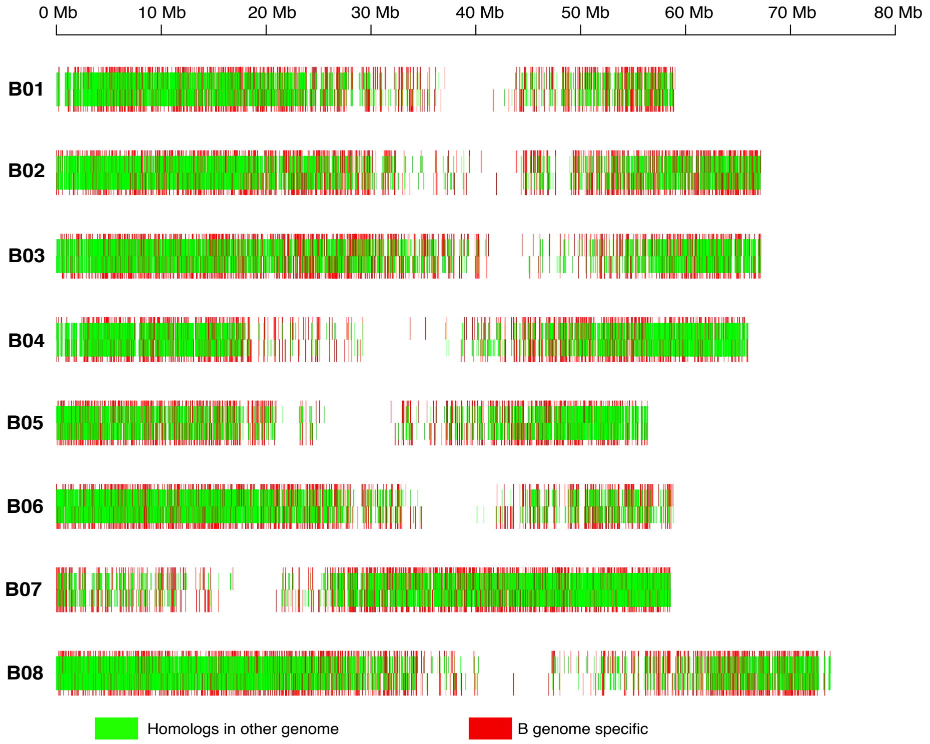

Figure S13. Gene density on *B. juncea* B genome pseudochromosomes. Green bars show genes that have orthologs in the A genome *B. juncea* and *A. thaliana*. Red lines represent the B genome-specific genes – (a) total predicted genes (19,193) on B01-B08 pseudochromosomes, (b) genes that were found to express in the transcriptome data (total 8,718).

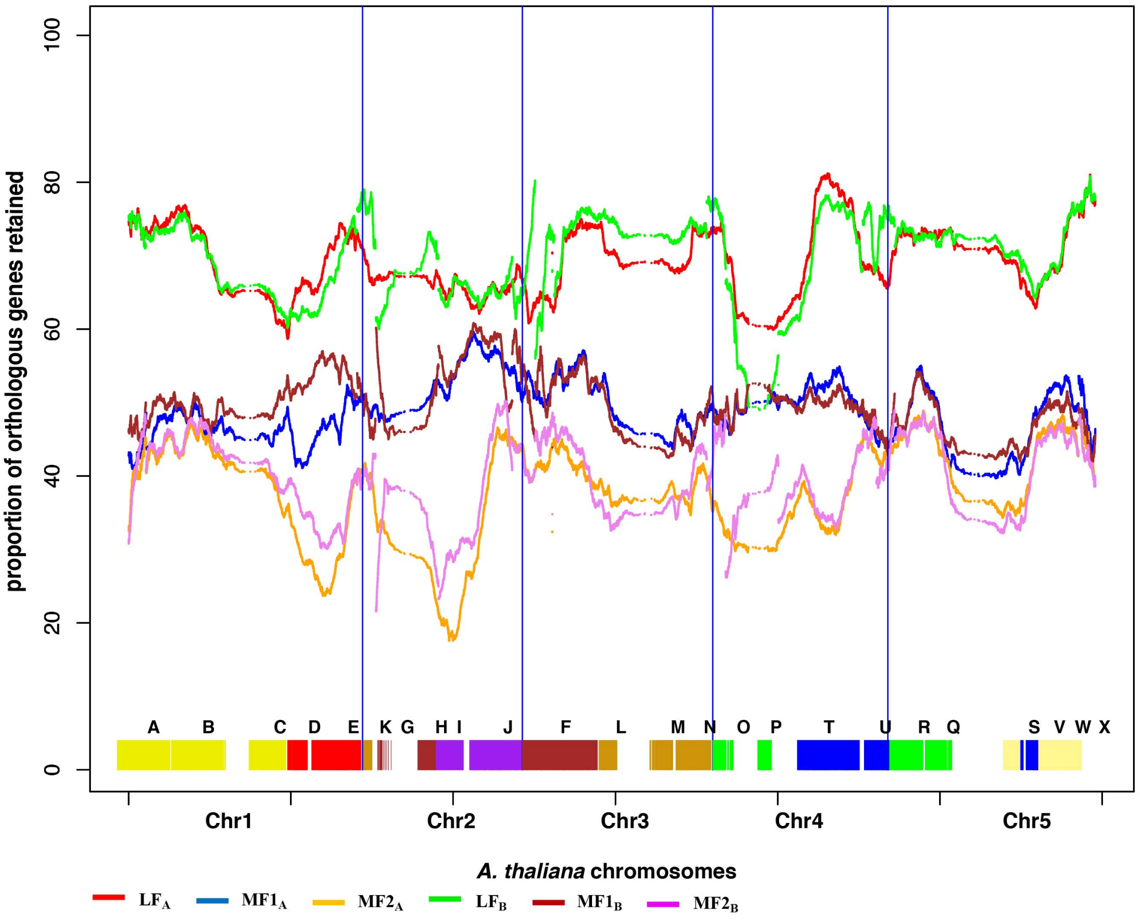

Figure S14. Proportion of orthologous genes retained in the A and B genomes of *Brassica juncea*. Gene retention/loss of the orthologous genes in the three paleogenomes (LF, MF1, and MF2) of the A and B genomes of *B. juncea* corresponding to *A. thaliana* A to X gene blocks. The x-axis shows the physical position of *A. thaliana* genes and gene blocks; the y-axis shows percentage orthologous genes retained in the three paleogenomes of the A and B genomes of *B. juncea*.

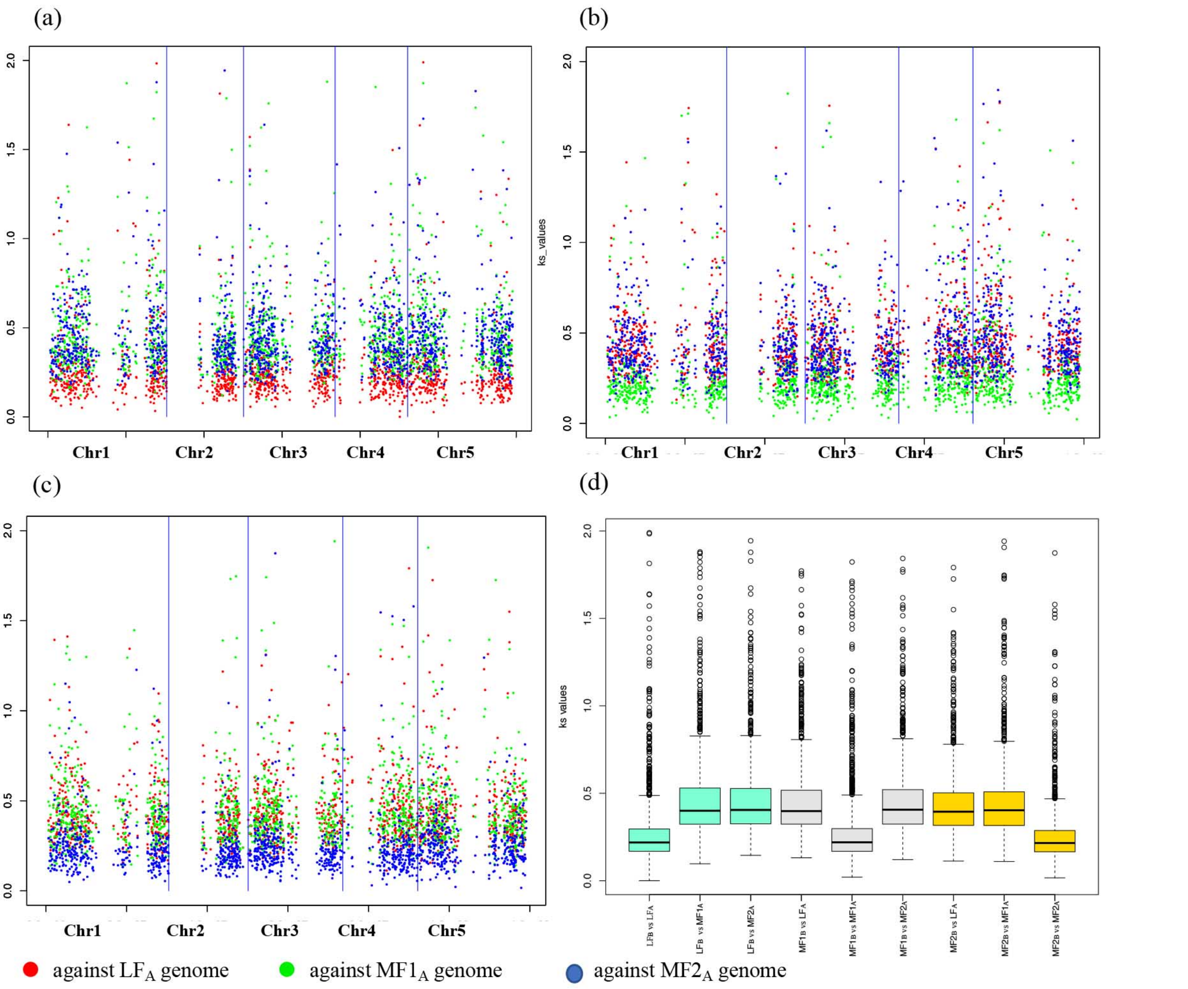

Figure S15. Relationship of the three paleogenomes(LF, MF1, and MF2) of the A and B genomes of *Brassica juncea* based on Ks values in 1,428 genes present as a single copy in *A. thaliana* and three copies each in the A and the B genomes. Ks values were calculated between (a) LF (b) MF1 and (c) MF2 region genes of the B genome against the three syntenic genes in the A genome. The positions of the genes have been arranged based on their physical position on the *A. thaliana* chromosomes (d) box and whisker plot showing inter-relationship of the three paleogenomes constituting the A and B genomes of *Brassica juncea*.

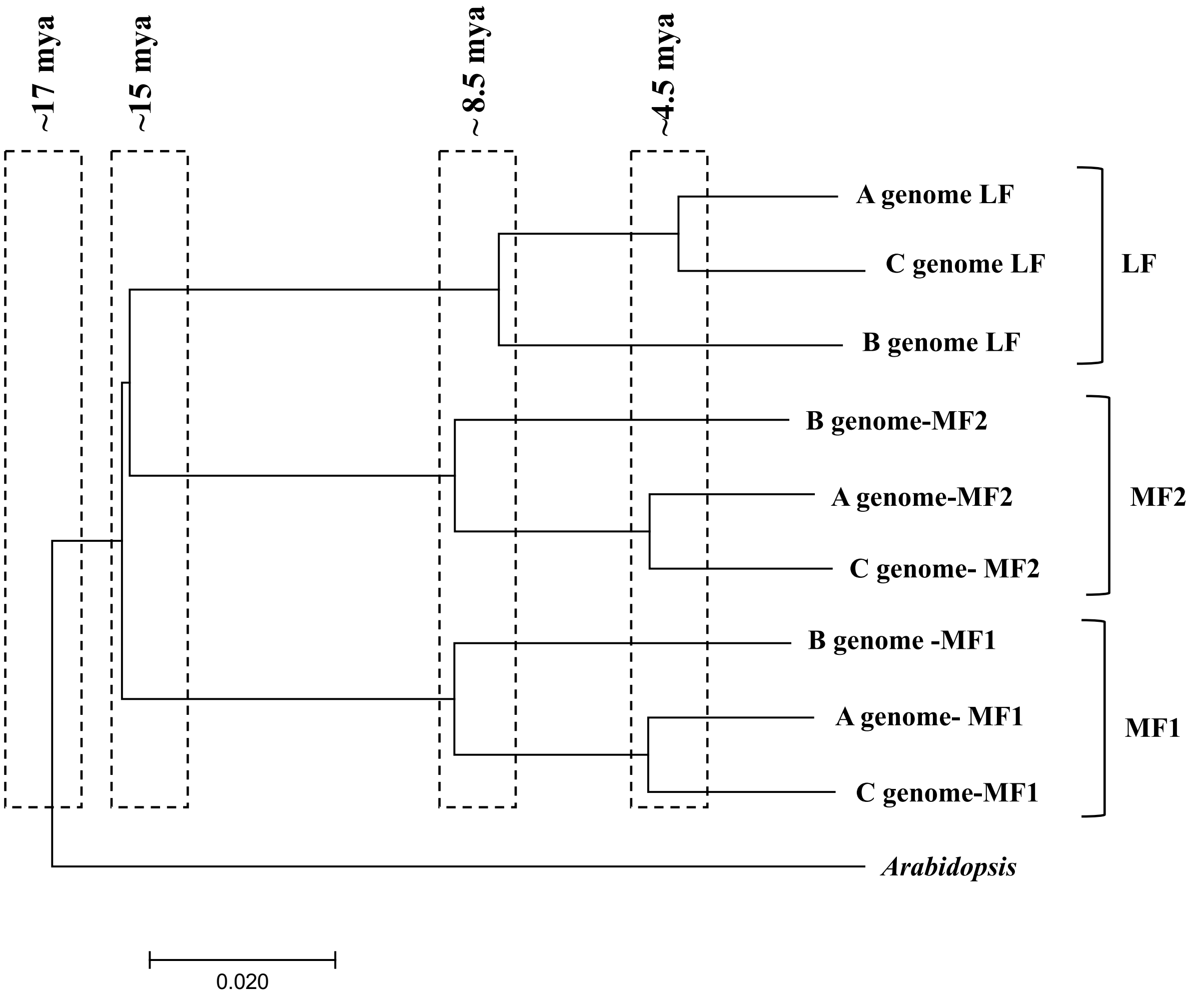

Figure S16. Neighbour-joining tree based divergence analysis of the constituent paleogenomes of the A, B, and C genomes as compared to *A. thaliana*. The tree was constructed based on the distances estimated from synonymous substitutions in the concatenated CDS sequences of 1,428 *Arabidopsis* genes that have been retained as three orthologs in the A and B genomes of *Brassica juncea* and the C genome of *B. oleracea*. A and B genome paralogs diverged around 15MYA, whereas the homoeologs diverged ~8.5 MYA; the A and C genome homoeologs diverged around ~4.5 MYA.

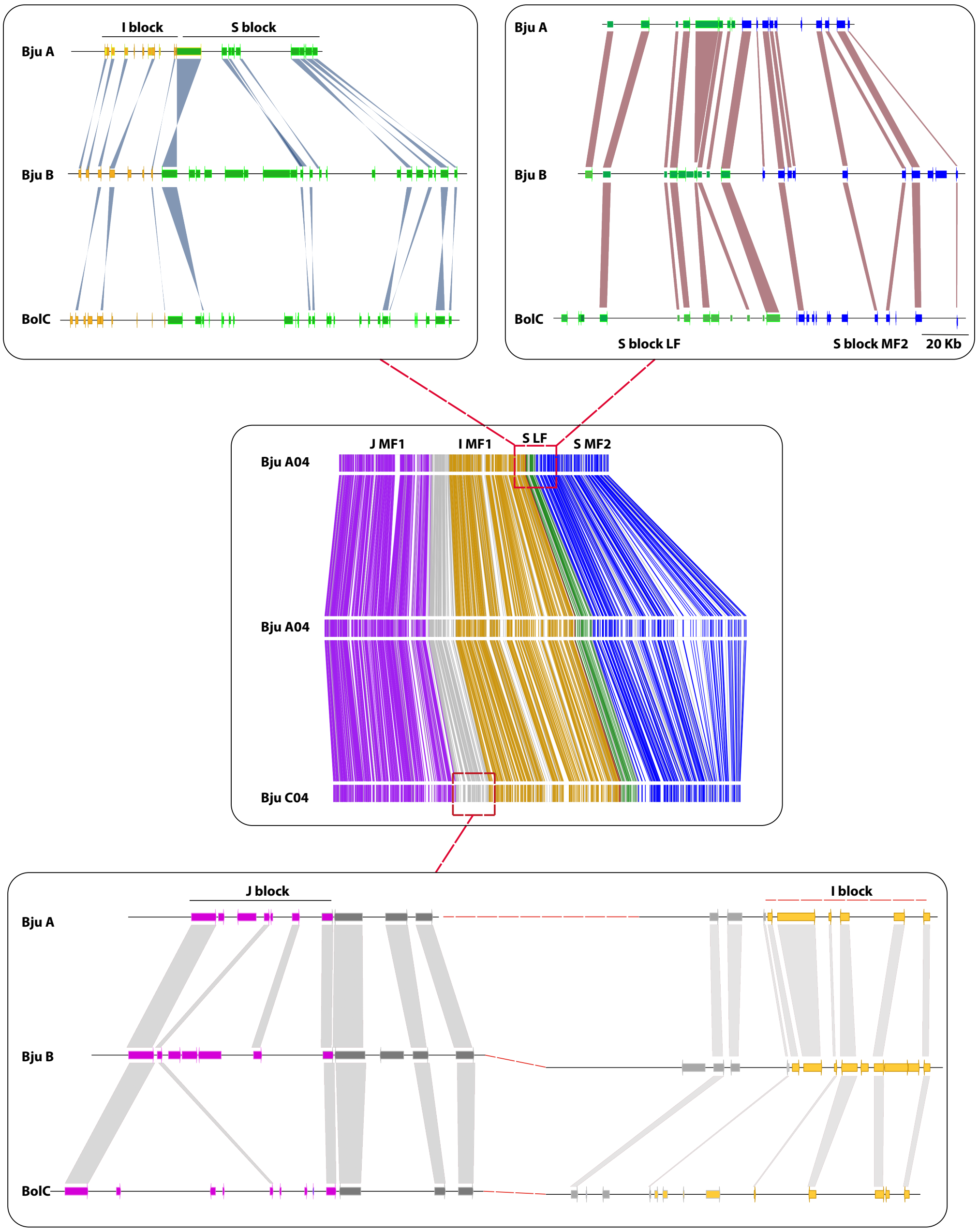

Figure S17. Gene collinearity and junction analysis of the the gene block association J_{MF1}-I_{MF1}-S_{MF2}-S_{LF} in the BjuA, BjuB and BolC genomes. Same gene block junctions were identified in all the three analyzed genomes. The block junction data suggests a common origin for the A, B and C genomes.
