# Supplementary material for: A chromosome‐scale assembly of allotetraploid Brassica juncea (AABB) elucidates comparative architecture of the A and B genomes
Source: Plant Biotechnol J. 2020 Dec 30;19(3):602–14. doi: 10.1111/pbi.13492 (PMC7955877; doi:10.1111/pbi.13492)
Supplement: Supplementary file 7 — File S1. Transcriptome assembly of Brassica juncea Varuna and Brassica nigra Sangam. [file PBI-19-602-s005.docx]

**File S1 Transcriptome assembly of *Brassica juncea* and *Brassica*** ***nigra***

***Brassica juncea* transcriptome sequencing**

For generating full-length transcriptome sequences, PacBio based sequencing (Iso-seq) was carried out. For validating the predicted genes, Illumina based short-reads from three different earlier studies (Paritosh et al., 2014; Sharma et al., 2015; Bhardwaj et al., 2015) were also mapped on the assembled chromosomes.

RNA was isolated from the pooled sample of the seedling, leaf, inflorescence with developing siliqua and seed as described in the materials and methods. The pooled RNA sample was considered to represent most of the expressed genes in *the B. juncea* plant. Two different transcriptome libraries, one in the size range of 0.5-1 kb and the other one of 1-2 kb, were developed and sequenced separately using P6-C4 chemistry on the PacBio RSII system. A total of 551,943 reads on inserts (ROIs) were generated ­– 182,848 from 0.5-1 kb library and 369,095 from 1-2 kb size library. After filtering the sequences for the presence of 5´ adapter reads, poly-A reads, chimeric reads – 114,068, and 176,916 full-length non-chimeric reads were obtained for 0.5-1 kb and 1-2 kb libraries, respectively.

Finally, clustering and polishing yielded 42,618 and 70,604 consensuses isoforms, of which 27,436 and 43,366 were high-quality isoforms that were obtained with 0.5-1 kb and 1-2 kb size libraries, respectively (Supplementary File 1 – Table1).

**SF1-Table 1 Statistics of PacBio based transcriptome sequencing of *B. juncea* Varuna**

| **Classification of reads of Insert 0.5-1 kb 1-2 kb** | | |
| --- | --- | --- |
| Number of reads of insert | 182,848 | 369,095 |
| Number of five prime reads | 132,278 | 243,413 |
| Number of three prime reads | 138,522 | 245,774 |
| Number of poly-A reads | 135,760 | 232,429 |
| Number of filtered short reads | 19,226 | 12,394 |
| Number of non-full-length reads | 48,948 | 176,402 |
| Number of full-length reads | 114,674 | 180,299 |
| Number of full-length non-chimeric reads | 114,068 | 176,916 |
| Average full-length non-chimeric read length | 822 | 1,347 |
| **Cluster stats** | | |
| Number of consensus isoforms | 42,618 | 70,604 |
| Number of polished, high-quality isoforms | 27,436 | 43,366 |
| Number of polished low-quality isoforms | 15,182 | 27,238 |
| Average consensus isoforms read length | 873 | 1,459 |

The obtained consensus isoforms were mapped on the assembled *B. juncea* genome sequences using GMAP software (Wu and Watanabe, 2005). Out of 113,222 consensus isoforms, 104,353 could be mapped on the reference genome, representing 35,423 genes in the transcriptome dataset.

In addition to the PacBio based transcriptome sequences, Illumina based short read transcriptome sequences from three of the previous studies on *B. juncea* were mapped on the assembled genome sequence (Supplementary File 1 – Table 2). Short read PE sequences were taken from SRR1822192 (Sharma et al., 2015), SRR1822193 (Sharma et al., 2015), SRR1718914 (Bhardwaj et al., 2015), SRR1718916 (Bhardwaj et al., 2015), SRR1718918 (Bhardwaj et al., 2015), SRR1269499 (Paritosh et al., 2014) were mapped on the assembled genome using STAR aligner (Dobin et al., 2013). Unique reads that mapped to each of the genes were calculated, and genes with more than 10 mapped reads were designated as expressed. A total of 79,108 genes were found to be represented in the transcriptome dataset, of which 37,256 genes belonged to the A genome, and 41,853 genes belonged to the B genome.

**SF1-Table 2 Details of previous transcriptome studies on *B. juncea***

| **SRR number** | **Tissues** | **Conditions** | ***B. juncea* variety** | **Genes represented** | **Ref.** |
| --- | --- | --- | --- | --- | --- |
| SRR1269499 | Seedling, stem, leaf, pod, developing inflorescence | Field Grown | Varuna | 67,198 | Paritosh et al., 2014 |
| SRR1718914 | Seedling | Normal growth | Varuna | 58,816 | Bhardwaj et al., 2015 |
| SRR1718916 | Seedling | Temperature stress | Varuna | 55,607 | Bhardwaj et al., 2015 |
| SRR1718918 | Seedling | Drought stress | Varuna | 55,348 | Bhardwaj et al., 2015 |
| SRR1822192 | Seedling | Normal growth | CS52 | 55,760 | Sharma et al., 2015 |
| SRR1822193 | Seedling | Salt stress | CS52 | 54,950 | Sharma et al., 2015 |

***B. nigra* transcriptome sequencing**

*Brassica nigra* line BnSDH-1 was used for RNA sequencing. RNA was isolated from the seedling and young inflorescence tissues using the procedures described in the Materials and Methods section. Paired-end (PE) libraries (2x101bp) were sequenced using GA IIx sequencer (Illumina); a total of 117,766,948 raw sequence reads were produced. These sequences were filtered to remove low-quality reads, which had a phread value <20 in 70% of the bases of the sequence. Further, 31 bases were trimmed from the 3′ end of each of the paired-end reads as the region invariably had a phread value of <25; this resulted in 73,864,802 high-quality PE sequences of 70 bp length for *B. nigra* (Supplementary File 1 – Table 3). Trinity based assembly of the filtered PE sequences generated 75,087 contigs with an N50 value of 1,333 bp. The longest read was 8,525 bp in size.

**SF1-Table 3 Statistics of Illumina based paired-end (PE) transcriptome reads of *B. nigra* Sangam**

| Library Type / Insert size | 250 bp |
| --- | --- |
| Total number of reads | 117,766,948 |
| Adapter trimmed reads | 115,207,472 |
| Quality filtered (Q<30) reads | 100,115,158 |
| Base trimmed paired- end reads | 73,864,802 |
| Clean pre-processed reads | 73,864,802 |
| % GC | 45 |
| Data in Mb | **1074.1** |

Additionally, a total of 152,075 reads were generated with Roche 454 (GS FLX titanium) based sequencing of *B. nigra* with an N50 value of 108 bp. Newbler based assembly of the raw reads was carried out with 100 bp overlap length with >90% identity; this yielded 25,798 isotigs with an N50 value of 1,244 bp (Supplementary File 1- Table 4). While Illumina based sequencing validated 35,556 of the 46,227 genes predicted in the genome assembly of *B. nigra* Sangam, 454 based sequencing could identify only 21,964 genes. Both the assemblies taken together validated 35,819 of the predicted genes.

**SF1-Table 4 Statistics of the transcriptome assembly of *B. nigra* Sangam**

|  | **Roche sequencing**  **Newbler assembly** | **Illumina sequencing Trinity assembly** |
| --- | --- | --- |
| Number of contigs | 25,798 | 75,087 |
| Total size of contigs | 26,752,765 | 67,340,324 |
| Longest contig | 6,108 | 8,525 |
| Shortest contig | 77 | 224 |
| Number of contigs > 1K nt | 10,664 | 24,758 |
| Mean contig size | 1,037 | 897 |
| Median contig size | 871 | 632 |
| N50 contig length | 1,244 | 1,333 |
| L50 contig count | 7,372 | 16,540 |
| contig %A | 27.04 | 28.34 |
| contig %C | 23.18 | 21.52 |
| contig %G | 22.36 | 21.91 |
| contig %T | 27.42 | 28.23 |
| contig %N | 0 | 0 |
| Contig %non-ACGTN | 0 | 0 |

**Additional references**

Wu T.D. and Watanabe C.K. (2005) GMAP: a genomic mapping and alignment program for mRNA and EST sequences. Bioinformatics. **21**, 1859-75
